# Genome-Based Molecular Diversity of Extended-Spectrum *β*-Lactamase-Producing *Escherichia coli* From Pigeons in China

**DOI:** 10.1155/2024/1828830

**Published:** 2024-10-01

**Authors:** Shuangyu Li, Xinshuai Liu, Haoyu Zhao, Yuhua Zhang, Zheng Lu, Juan Wang, Ruichao Li, Peng Xie, Yibin Hu, Caiyuan Zhou, Qian Mao, Leilei Sun, Shanshan Li, Wenhui Wang, Fang Wang, Xinyu Liu, Tiantian Liu, Wei Pan, Chengbao Wang

**Affiliations:** ^1^ College of Veterinary Medicine Northwest A&F University, Yangling 712100, Shaanxi, China; ^2^ DSM (Netherlands), Heerlen 6411, Netherlands; ^3^ College of Veterinary Medicine Yangzhou University, Yangzhou 225009, Jiangsu, China; ^4^ Jiangsu Key Laboratory of Immunity and Metabolism Jiangsu International Laboratory of Immunity and Metabolism Department of Pathogen Biology and Immunology Xuzhou Medical University, Xuzhou 221004, Jiangsu, China

**Keywords:** antimicrobial resistance genes, ESBL-ECs, multidrug resistance, pigeons, whole genome sequencing

## Abstract

Extended-spectrum *β*-lactamase-producing *Escherichia coli* (ESBL-EC) strains present a significant menace to the well-being of both animals and humans. However, limited information is available regarding their profiles in pigeons. Using a combination of whole genome sequencing, drug susceptibility testing, and bioinformatics analysis, we examined the genomic features and epidemiology of 95 ESBL-EC strains (41 racing and 54 meat pigeons) that were isolated from 11 Chinese cities. These strains belonged to seven phylogenetic groups (A, B1, B2, C, D, E, and F). Moreover, these isolates have 51 serotypes, including several pathogenic ones (e.g., O51, O8, O4, O25, and O6). Notably, two high-risk clones, ST131 O25:H4, were found in racing pigeons and were responsible for the worldwide outbreaks of highly pathogenic and multidrug-resistant (MDR) *E. coli* infections. In addition, we found 41 multilocus sequence typing types, of which the dominant types were ST155, ST20, ST1011, and ST1196. In total, 91 isolates (95.79%) showed MDR, while eight isolates (8.42%) showed resistance to up to eight classes of antibiotics. Furthermore, we identified a series of ESBL genes in these isolates, including *bla*_CTX-M_, *bla*_TEM_, *bla*_OXA_, *bla*_LAP_, and *bla*_CMY_. Also, 50 other antibiotic resistance genes (ARGs) were accompanied by the carriage of 33 plasmid replicon types, facilitating the horizontal spread of ARGs. Interestingly, three *mcr-1*, four *mcr-1.1*, and one *tet*(X4) were found in isolates of meat pigeons, and it was possible to successfully transfer the plasmids containing *tet*(X4) and *mcr-1.1* to *E. coli* C600. In summary, this work presents the complexity of MDR profiles, plasmid profiles, and multiple typing profiles of Chinese ESBL-EC isolates of pigeon origin for the first time. The thorough investigation of ESBL-EC in pigeons presented in this work suggests that racing and meat pigeons are significant ARGs reservoirs.

## 1. Introduction

Pigeon farming is widely practiced and popular globally. Antibiotic resistance genes (ARGs) present in meat pigeons, a frequently consumed food, have the potential to be transferred directly to humans or animals along the food chain [1]. By 2021, China has around 40.805 million pairs of meat pigeons and 602 million squabs [2]. Notably, the racing pigeon industry in China has also seen rapid development. The average annual total number of racing pigeons has reached more than 25 million [3]. During training and competitions, racing pigeons can fly hundreds of kilometers. Studies have shown that pigeon feces during flights can be potential sources and vectors for the spread of genes and bacteria resistant to antibiotics, thereby heightening the risk of multidrug-resistant (MDR) *Escherichia coli* dispersing throughout areas from racing pigeons [4]. In addition, importing breeding pigeons from abroad is possible to increase the flow of ARGs between continents. Therefore, pigeons might serve as a key reservoir for the spread of possible antimicrobial resistance (AMR) in humans.

The quick rise in AMR rates is one of the most significant challenges the globe is currently facing. MDR bacteria also pose a major threat to the health of both humans and animals [5]. *E. coli* is present in a diverse array of hosts and is regarded as a valuable indicator of the prevalence of AMR across various populations and animal groups [6]. Additionally, ARGs can be acquired by *E. coli* and passed on to other bacteria [7]. The swift expansion of MDR in *E. coli*, particularly the rising incidence of extended-spectrum *β*-lactamases (ESBLs), has adversely affected the efficacy of treatment and elevated morbidity and mortality in both humans and animals [8].

*β*-Lactamases have the ability to hydrolyze the beta-lactam cyclic amide bond, rendering beta-lactamase antibiotics inactive [9]. It is categorized into distinct classes according to their sequences of amino acids, including Class A (e.g., CTX-M, TEM, and SHV enzymes), Class C (e.g., CMY, DHA, and ACT enzymes), and Class D (e.g., OXA enzymes) *β*-lactamases. Furthermore, genes that code for *β*-lactamases are typically referred to as *bla*, then the name of the particular enzyme (e.g., *bla*_CTX-M_) [7]. The *bla* gene responsible for encoding *β*-lactamase is commonly found on plasmids. Plasmid-associated integrons are mobile genetic elements capable of capturing various ARGs and forming complex operons, which can aid in the horizontal gene transfer (HGT) that spreads AMR [7]. Furthermore, some plasmids carry additional ARGs in addition to the *bla* genes. Co-selection and long-term presence of plasmids carrying the *bla* gene are promoted even in the absence of *β*-lactam antibiotics [10]. This is why ESBLs are regarded as pivotal in the spread of MDR in *E. coli*.

ESBL-producing *E. coli* (ESBL-EC) strains are omnipresent, which can spread in humans [11], food animals [12, 13], and even in fruits [14], vegetables [14], and environments [15]. However, at present, only a handful of studies have examined the partial ARGs in ESBL-EC isolated from pigeons. For instance, a recent research has identified ESBL-EC in pigeons from South Brazil [16], Kerman in southeastern Iran [17], Qingdao in China [18], Lisbon in Portugal [19], and Slovakia [20]. However, these studies only employed polymerase chain reaction (PCR) to identify a limited set of ARGs.

In the present study, using a combination of drug susceptibility testing and whole genome sequencing (WGS), we investigated the phenotypic and genotypic MDR profiles, plasmid replicon profiles, multilocus sequence typing (MLST) types, phylogenetic groups, and serotypes of pigeon-derived ESBL-EC isolates in 11 randomly selected cities across seven provinces in China. To the best of our knowledge, this study offers the first thorough report on a wide range of ESBL-EC isolates in Chinese pigeons, suggesting the potential role of pigeons as carriers of ARGs entering the human population.

## 2. Materials and Methods

### 2.1. Sample Collection and ESBL-EC Isolation

**Figure 1:**
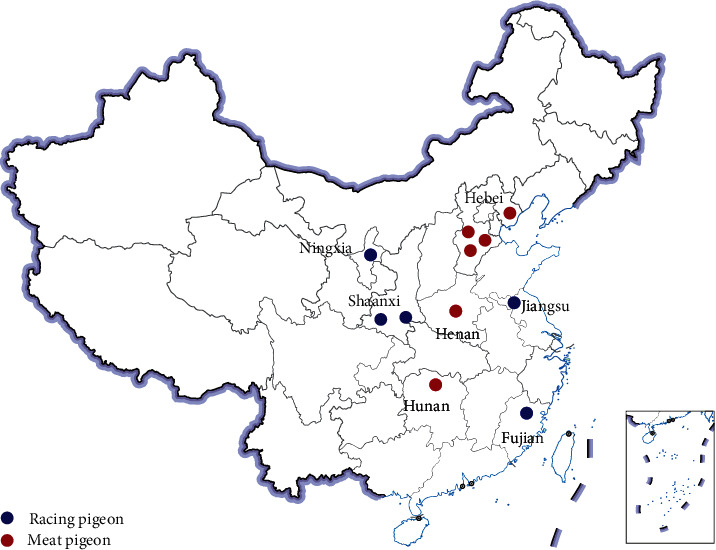
The geographical distribution of ESBL-EC strains isolated from racing pigeons and meat pigeons in 11 cities of 7 provinces in China. Blue represents racing pigeons, and red represents meat pigeons.

Between May and August 2022, a sum of 147 cloacal swab samples from racing pigeons (*n* = 80) and meat pigeons (*n* = 67) were collected randomly on farms from 11 cities in seven provinces in China, including four cities in Hebei province, two cities in Shaanxi, and one city in Ningxia, Henan, Jiangsu, Hunan, Fujian, respectively. These provinces cover the northern, northwestern, central, eastern, central-southern, and southeastern coastal regions of China (Figure 1).

Cloacal swab specimens were aseptically gathered in sterile receptacles, stored in a cryogenic incubator (4°C), and promptly dispatched to the laboratory. The samples were received, placed in 3 mL of lysogeny broth (LB), and vigorously agitated (180 r/min) for the duration of the enrichment process at 37°C. After that, they were cultivated on MacConkey agar. Following 18–24 h of incubation at 37°C, a single pink colony was chosen and subcultured onto eosin–methylene blue agar, where it underwent the previously mentioned incubation. Colonies with greenish metallic tints were thought to be probable *E. coli* isolates; however, 16S rDNA gene sequence analysis and Gram staining verified the identity of *E. coli*.

Following the criteria published by the Clinical and Laboratory Standards Institute (CLSI, 2020), MacConkey agar mixed with cefotaxime (4 *μ* g/mL) was used to screen *E. coli* isolates in order to identify ESBL producers. The double disk synergy test involving ceftazidime (30 *μ* g), cefotaxime (30 *μ* g), ceftazidime-clavulanic acid (30 *μ* g/10 *μ* g), and cefotaxime-clavulanic acid (30 *μ* g/10 *μ* g) further supported these presumed ESBL-EC. As quality control strains, ESBL-negative *E. coli* strain ATCC 25922 and ESBL-positive *Klebsiella pneumoniae* strain ATCC 700603 were employed.

### 2.2. Antimicrobial Susceptibility Testing

According to the CLSI guidelines (CLSI, 2020), Thermo Scientific Sensititre GN4F Gram-negative bacteria drug sensitivity plates (Thermo Fisher Scientific, Waltham, MA) were used to test for MIC to 23 antibiotics of nine classes using the broth microdilution method. They were cephalosporins (ceftazidime [CAZ, 1–16 *μ* g/mL], cefepime [FEP, 4–32 *μ* g/mL], ceftriaxone [CRO, 0.5–32 *μ* g/mL] and cefazolin [CZO, 1–16 *μ* g/mL]), carbapenems (meropenem [MEM, 0.5–8 *μ* g/mL], ertapenem [ETP, 0.25–8 *μ* g/mL] and imipenem [IMP, 0.5–8 *μ* g/mL]), monobactams (aztreonam [ATM, 1–16 *μ* g/mL]), aminoglycosides (gentamicin [GEN, 2–8 *μ* g/mL], tobramycin [TOB, 2–8 *μ* g/mL] and amikacin [AMK, 8–32 *μ* g/mL]), tetracyclines (tigecycline [TIG, 1–8 *μ* g/mL], minocycline [MNO, 1–8 *μ* g/mL] and tetracycline [TET, 4–8 *μ* g/mL]), quinolones (levofloxacin [OFX, 1–8 *μ* g/mL] and ciprofloxacin [CIP, 0.5–2 *μ* g/mL]), sulfanilamides (trimethoprim/sulfamethoxazole [SXT, 2/38−4/76 *μ* g/mL]), penicillins (ampicillin [AMP, 8–16 *μ* g/mL], piperacillin [PIP, 16–64 *μ* g/mL], piperacillin/tazobactam [TZP, 8/4−128/4 *μ* g/mL], ticarcillin/clavulanic acid [TCC, 8/2−64/2 *μ* g/mL] and ampicillin/sulbactam [SAM, 4/2−16/8 *μ* g/mL]) and nitrofuran (nitrofurantoin [NIT, 32–64 *μ* g/mL]). The *E. coli* ATCC 25922 was used for quality control.

### 2.3. Detection of Phylogenetic Groups

The new quadruplex phylogroup assignment approach of Clermont et al. [21] was used to identify the phylogenetic groups of 95 ESBL-EC isolates. In particular, the phylogenetic group affiliations (A, B1, B2, C, D, E, or F) of these isolates were determined by quadruple PCR using the genes *chuA*, *yjaA*, *arpA*, and the DNA fragment *TspE4.C2*.

### 2.4. WGS

In total, 95 ESBL-EC strains were subjected to WGS so as to obtain genotypic characterization. Using a kit from TianGen (Beijing, China), the genomic DNA from these isolates was extracted, and next-generation sequencing was performed on an Illumina platform using the PE150 technique (Novogene, Tianjin, China). Next, we combined the Illumina raw readings using SPAdes version 3.14.0 (http://bioinf.spbau.ru/spades).

### 2.5. Bioinformatics Analysis

To assess the quality of genome assembly, QUAST (http://quast.sourceforge.net/quast) was employed. The assembled sequences were automatically annotated using the rast (https://rast.nmpdr.org/rast.cgi) [22] and prokka (https://github.com/tseemann/prokka) [23]. Serotypes, ARGs, plasmid replicon types, insertion sequences (IS), MLST, and FimH/FumC were identified by the Center for Genomic Epidemiology (http://www.genomicepidemiology.org/) [24–26]. BioNumerics 7.6 was used to construct the visualization of the MLST allelic profiles of the *E. coli* core genome. Furthermore, Roary and FastTree, which use the maximum likelihood method, were used to construct an evolutionary tree. The phylogenetic tree, which includes the discovered ARGs, MLST, FimH/FumC, and serotype, was visually shown in this study using the Interactive Tree of Life (https://itol.embl.de) web server.

### 2.6. Statistical Analysis

Using SPSS software version 19.0, *chi*-square tests were performed to ascertain the effect of different factors on the rates of AMR in *E. coli*. A probability value (*P*) less than 0.05 was deemed to indicate statistical significance.

### 2.7. Conjugation Assays

Conjugation tests were conducted using *E. coli* C600 (rifampin-resistant) as the recipient and isolates expressing the relevant resistance genes as donors in order to examine the transferability of *mcr-1*, *mcr-1.1*, and *tet*(X4). Donor and recipient cultures that reached 0.5 McFarland culture density were mixed at a 1:1 ratio, respectively. 0.1 mL of the mixed culture was applied onto LB agar plates and incubated at 37°C for 12 h. The bacterial cultures on the plates were collected and diluted 10-fold with sterile saline. Then 10 *μ* L of diluted bacterial solution was aspirated and inoculated onto the screening plate. We screened transconjugants carrying *mcr* on LB agar plates containing rifampicin and colistin and carrying *tet*(X4) on LB agar plates containing rifampicin and tigecycline. The concentrations of antibiotics used were as follows: rifampicin, 50 *μ* g/mL; colistin, 2 *μ* g/mL; tigecycline, 2 *μ* g/mL. The protocol outlined by Li et al. [27] was used to carry out conjugation transfer, and transconjugants were identified by PCR and corresponding resistance phenotyping. The PCR primers used in the conjugation assay are shown in Table S1.

## 3. Results

### 3.1. ESBL-EC and MLST Pattern Distribution

**Figure 2:**
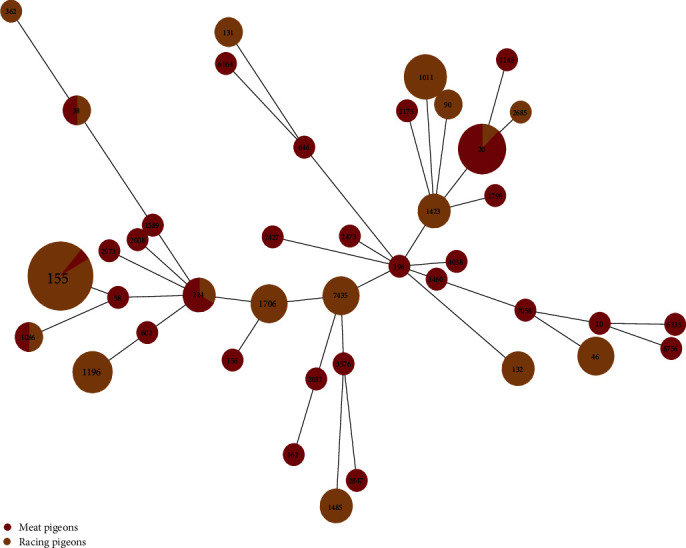
The distribution of MLST of ESBL-ECs. A distinct sequence type (ST) is represented by each circle, and the size of the circle indicates the number of isolates in that ST. The number of differentiating alleles is shown by the distance labels. Yellow represents racing pigeons, and red represents meat pigeons.

**Figure 3:**
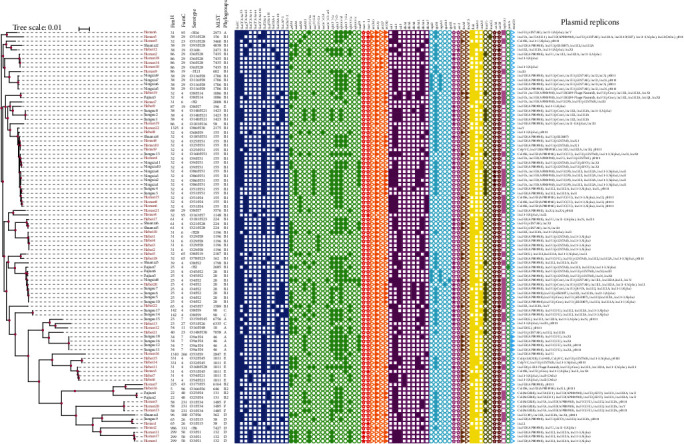
An evolutionary tree analysis of 95 ESBL-EC strains was conducted, encompassing phylogenetic groups, MLST, *fum*C-*fim*H types, serotypes, ARGs, and plasmid replicons. Strains labeled in red are meat pigeons, and black are racing pigeons.

Among 147 samples, 137 *E. coli* isolates were identified, and 95 of them were presumed as ESBL-EC, with meat pigeons accounting for 80.60% (54/67) and racing pigeons accounting for 51.25% (41/80) (Table 1). These strains were classified into 41 MLST types, with 31 types observed in meat pigeons and 15 types in racing pigeons. The most prevalent MLST types were ST155 (*n* = 18), ST20 (*n* = 8), ST1011 (*n* = 6), and ST1196 (*n* = 5). Interestingly, five MLST types, namely ST1086, ST155, ST224, ST20, and ST38, were shared between meat pigeons and racing pigeons. Among them, ST155 was dominated in the racing pigeons, while ST20 was more predominant in meat pigeons. Notably, the MLST types of meat pigeons (57.41%, 31/54) exhibited greater diversity, being widely distributed and dispersed. On the other hand, racing pigeons (31.71%, 13/41) had fewer and more concentrated MLST types (Figure 2). Phylogenetic analysis revealed that ST155 exhibited a widespread distribution spanning six provinces, and isolates of the same ST type were more closely related. In addition, two high-risk clones of ST131 were observed (Figure 3). Furthermore, according to CH typing, we identified 25 *fim*H alleles and 20 *fum*C alleles, which formed 36 CH types. FumC4/FimH32 was the most abundant type, accounting for 21.05%. Followed by FumC4/FimH25 and FumC6/FimH31, which account for 9.47% and 6.32%, respectively. The most prevalent *fum*H alleles among the 95 ESBL-EC isolates were C4 (49.47%, *n* = 47) and C29 (10.53%, *n* = 10), whereas the most prevalent *fum*H alleles were H32 (25.26%, *n* = 24), H31 (11.58%, *n* = 11), and H38 (10.53%, *n* = 10) (Figure 3).

### 3.2. Serotypes and Phylogenetic Grouping for the ESBL-EC Isolates

Seven phylogenetic groupings were identified from 95 ESBL-producing isolates, namely A (*n* = 8, 8.42%), B1 (*n* = 62, 65.26%), B2 (*n* = 4, 4.21%), C (*n* = 3, 3.16%), D (*n* = 7, 7.37%), E (*n* = 8, 8.42%), and F (*n* = 3, 3.16%) (Figure 3). Among which, B1 group was predominant both in racing and meat pigeons. It is worth noting that E and F were exclusively found in meat pigeons, while the remaining five phylogenetic groups were present in both meat and racing pigeons. Phylogenetic groups in meat pigeons (12.96%, 7/54) are more than in racing pigeons (12.20%, 5/41).

These ESBL-producing isolates were further characterized by their serotypes. A total of 51 serotypes were identified, with only the H antigen being detected in five genomes (Figure 3). From an overall perspective, the most frequently observed serotypes were O51 (*n* = 9), O8 (*n* = 8), O4 (*n* = 6), O25 (*n* = 5), O1 (*n* = 5), O9a (*n* = 4), O116 (*n* = 4), O29 (*n* = 4), and O6 (*n* = 4). There were 37 serotypes of meat pigeons, with O51 as the most common. There were 20 serotypes of racing pigeons, with O4 and O9a as the most common. O4:H2, O1:H5, O8:H14, and O9:H51 were shared between meat and racing pigeons. It is clear that the serotypes of isolates in meat pigeons (68.52%, 37/54) are more diverse compared to these in racing pigeons (48.78%, 20/41).

### 3.3. Phenotypic AMR Characteristics

**Figure 4:**
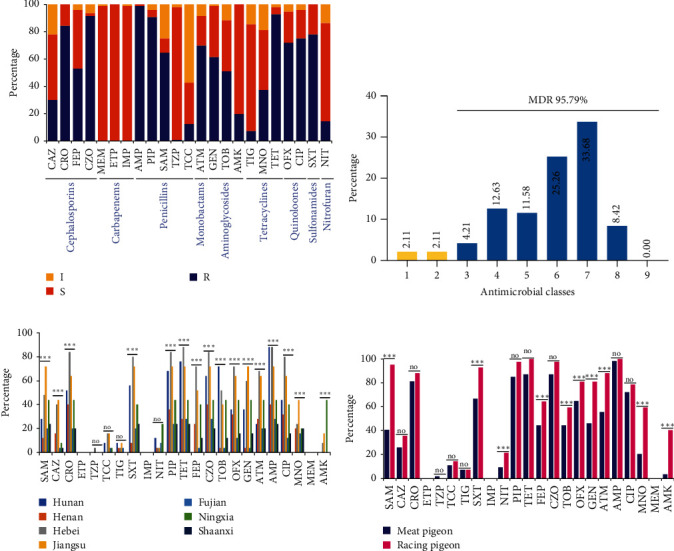
The AMR phenotypes of 95 ESBL-EC isolates in pigeons. (a) The column chart illustrates the AMR rates of *E. coli* isolates, with blue, yellow, and orange shading denoting resistant (R), intermediate (I), and susceptible (S) rates, respectively. (b) The prevalence of MDR among the 95 ESBL-EC isolates is depicted, with the isolates exhibiting MDR represented by the blue rate. The non-MDR isolates are indicated in yellow. (c) The resistance rates of ESBL-EC isolates from pigeons across various cities are depicted, with each color corresponding to a specific region: Hunan (blue), Henan (orange), Hebei (gray), Jiangsu (yellow), Fujian (light blue), Ningxia (green), and Shaanxi (dark blue). (d) The disparities in resistance rates between isolates from meat pigeons and racing pigeons across 23 antimicrobials are indicated, with  ^*∗∗∗*^, *P* < 0.001 signifying a statistically significant difference; “no” denotes no significant difference between the two groups (*P* > 0.05).

These isolates showed high resistance rates to several antibiotics, including ampicillin (AMP, 98.95%), tetracycline (TET, 92.63%), cefazolin (CZO, 91.58%), piperacillin (PIP, 90.53%), ceftriaxone (CRO, 84.21%), trimethoprim/sulfamethoxazole (SXT, 77.89%), ciprofloxacin (CIP, 74.74%), levofloxacin (OFL, 71.58%), and aztreonam (ATM, 69.47%). On the other hand, lower resistance rates to ceftazidime (CAZ, 29.47%), amikacin (AMK, 20.00%), nitrofurantoin (NIT, 13.68%), ticarcillin/clavulanic acid (TCC, 11.58%), tigecycline (TIG, 6.32%), and piperacillin/tazobactam (TZP, 0.01%) were observed in these isolates. Furthermore, these isolates showed high or even complete susceptibility to imipenem (IMP), meropenem (MEM), and ertapenem (ETP) (Figure 4a).

Most of the isolates (91, 95.79%) were found to be MDR bacteria, meaning they were resistant to three or more distinct kinds of antibiotics (Figure 4b). Except for carbapenems, every isolate showed resistance to at least one of the other eight families of antibiotics. Among the nine classes of antimicrobials evaluated, resistance to seven classes was the most prevalent (31, 32.63%). The combination involved resistance to cephalosporins, penicillins, monobactams, aminoglycosides, tetracyclines, quinolones, and sulfonamides. CAZ-CRO-CZO-CZX-AMP-PIP-ATM-SAM-GEN-MNO-TET-OFL-CIP-SXT was identified as commonly observed MDR categories. Interestingly, a notable number of isolates (8, 8.42%) displayed resistance to a combination of up to eight classes of antibiotics (Figure S1 and Table S2).

Significant differences were seen in the rates of AMR among ESBL-EC strains from various origins, with the exception of ETP, TZP, TCC, TIG, IMP, NIT, and MEM. Generally, the AMR rates of ESBL-EC isolated from Hebei, Jiangsu, and Hunan were higher compared to other cities, while strains from Fujian exhibited lower AMR rates. Moreover, strains from Jiangsu showed relatively high AMR rates for various antimicrobials (Figure 4c). Additionally, The AMR rates of meat and racing pigeon isolates are shown in Figure 4d. The ESBL-EC isolates in racing pigeons exhibited significantly higher resistance rates to SAM, SXT, NIT, FEP, TOB, OFX, GEN, ATM, MNO, and AMK antibiotics compared to those isolates in meat pigeons but no significant difference in CAZ, CRO, TZP, TCC, TIG, and AMP (Figure 4d). In summary, the AMR of ESBL-EC strains is quite serious in racing pigeons and meat pigeons regardless of where the samples come from.

### 3.4. Carriage of ESBL Genes and Other ARGs

The majority of the ESBL genes belonged to *bla*_CTX-M_ (83.16%, *n* = 79), followed by *bla*_TEM_ (67.37%, *n* = 64), *bla*_OXA_ (27.37%, *n* = 26), *bla*_LAP_ (12.63%, *n* = 12), and *bla*_CMY_ (2.11%, *n* = 2). Notably, all ESBL-EC did contain the *bla*_Ec_. Among the *bla*_CTX-M_, the subtypes detected were *bla*_CTX-M−55_ (54.74%, *n* = 52), *bla*_CTX-M−65_ (21.05%, *n* = 20), *bla*_CTX-M−14_ (9.47%, *n* = 9), *bla*_CTX-M−64_ (2.11%, *n* = 2), and *bla*_CTX-M−121_ (1.05%, *n* = 1). Among the 79 isolates carrying the *bla*_CTX-M_, only 15 carried this gene alone, while the remaining 64 isolates carried at least one additional *bla* simultaneously. Remarkably, five isolates carried a combination of four different *bla* concurrently (Figure 3).

In addition to ESBL genes, the 95 isolates included a total of 50 additional ARGs. Among these ARGs, 17 genes were widespread and found in at least 25 of the 95 isolates. It is noteworthy that we found four isolates carrying *mcr-1.1* and three isolates expressing *mcr-1*, one of which also carried *tet*(X4). Notably, the seven isolates were all isolated from meat pigeons. Conjugation assays were performed on these seven strains, and the results showed that three strains carrying *mcr-1* did not express phenotypic resistance. Additionally, conjugation was utilized to successfully transfer the plasmids containing *tet*(X4) and *mcr-1.1* from four strains into *E. coli* C600. The *mcr-1.1*-bearing plasmid transfer frequencies were (1.33 ± 0.12) × 10^−8^, (2.11 ± 0.52) × 10^−6^, (9.4 ± 0.1) × 10^−7^, and (1.84 ± 0.63) × 10^−11^, respectively. The transfer of *tet*(X4)-bearing plasmid was at a frequency of (4.9 ± 0.33) × 10^−9^ (Figure S2). In addition, the following ARGs were detected in at least 20 strains. It is consist of aminoglycosides (*aadA1* [*n* = 41], *aadA2* [*n* = 27], *aph*(*6*)-*Id* [*n* = 64], *aph*(3″)-*Ib* [*n* = 55], *aph*(*3′*)-*Ia* [*n* = 41], *aph*(*3′*)-*IIa* [*n* = 28], *aac*(*3*)-*IVa* [*n* = 25], and *aph*(*4*)-*Ia* [*n* = 25]), tetracyclines (*tet*(A) [*n* = 86], *tet*(M) [*n* = 29]), sulfonamides (*sul2* [*n* = 62], *sul3* [*n* = 32], and *dfrA14* [*n* = 46]), quinolones (*qnrS1* [*n* = 55]), rifampins (*arr-2* [*n* = 26]), fosfomycins (*fosA3* [*n* = 21]), chloramphenicols (*cmlA5* [*n* = 21] and *floR* [*n* = 78]), azithromycins (*mph*(A) [*n* = 32]), lincosamides (*lnu*(F) [*n* = 24]) (Figure 3).

### 3.5. Plasmid Replicon Profiles Associated With AMR

**Figure 5:**
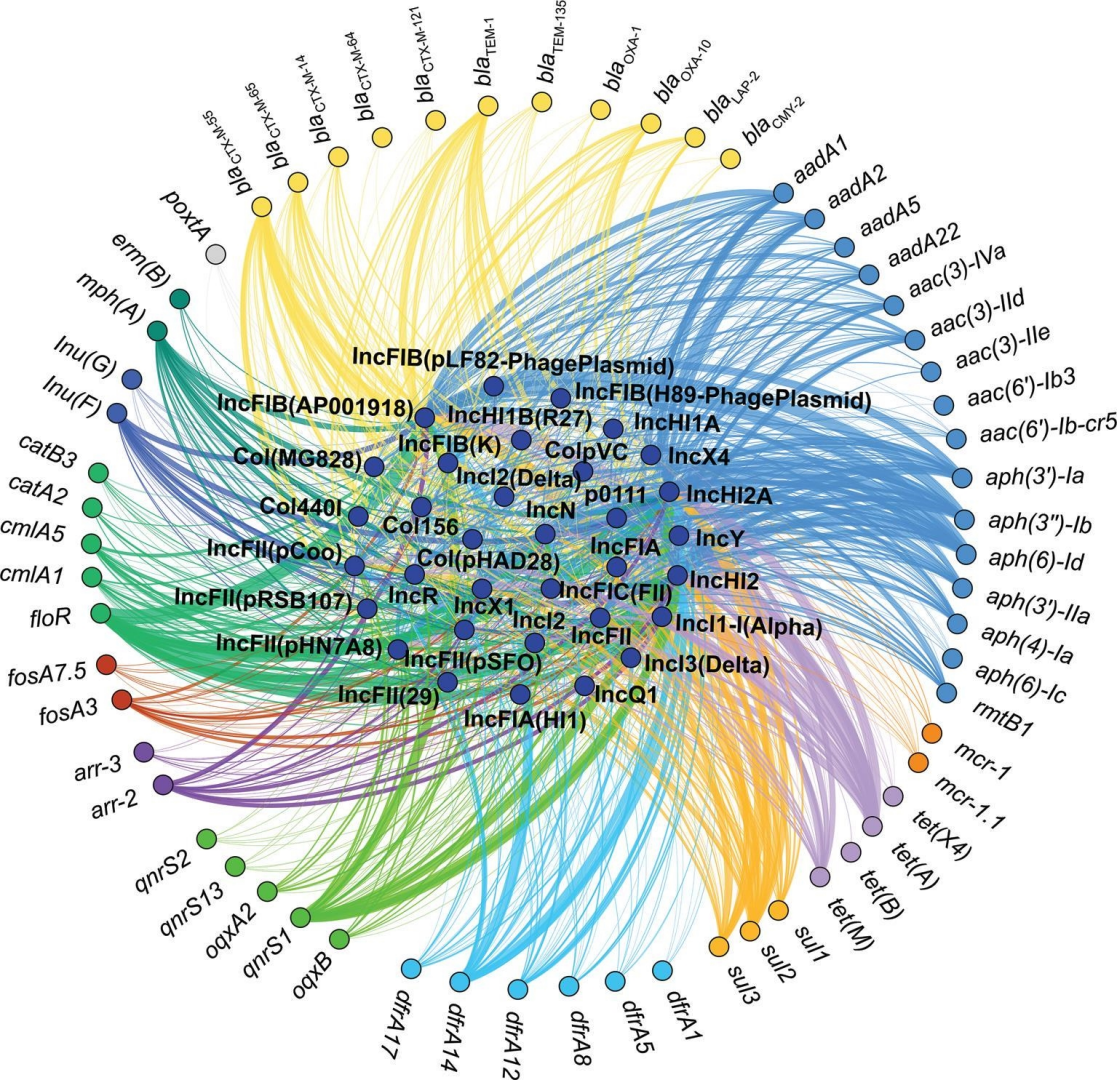
The ARGs and replicons co-occurrence network in 95 ESBL-EC isolates. Nodes represent genes, and their colors signify the antimicrobial class to which the AMR genes belong.

Among 95 isolates, 33 plasmid replicon types were predicted, while only one strain did not harbor any replicon. Even up to nine plasmid replicons were observed in one isolate, which notably also carried both *mcr-1.1* and *tet*(X4). Overall, the top six types were IncFIB(AP001918), Incl1-I(Alpha), IncHI2, IncHI2A, IncFII(pHN7A8), and p0111 (Table S3), with the percentage of 71.58%, 51.58%, 44.21%, 44.21%, 29.47%, and 29.47%, respectively. We found that IncHI2 and IncHI2A always appear in identical isolates at the same time. Most of the strains carrying the same replicons carried similar ARGs (Figure 3). The co-occurrence of ARGs and plasmid replicon types is depicted in Figure 5. A sum of 62 ARGs was carried by 33 types of replicon, and most were detected in IncFIB(AP001918), Incl1-I(Alpha), IncHI2, and IncHI2A.

## 4. Discussion

Animals are transmission sources/vectors of AMR bacteria and ARGs and are important hosts for ESBL-EC isolates [28]. However, there is only limited information in the profile of ESBL-EC isolates in pigeons, and previous studies only identified a limited number of ARGs using PCR techniques [16–20]. Here, using a combination of drug susceptibility testing and WGS, we monitored the MDR and sequence characterization of ESBL-EC strains in meat pigeons and racing pigeons in China. We found all ESBL-EC strains contained ESBL genes. Moreover, we identified *mcr-1* and *tet*(X4), the last line of defense against antibiotics' gene, as well as the ST131 high-risk clone. Our finding highlights the importance of monitoring extensive drug resistance in pigeons. Our results emphasize the significance of keeping an eye on pigeons' widespread AMR.

The ST profiles of 95 ESBL-EC strains were analyzed using MLST. We identified a total of 41 STs, with 31 in meat pigeons and 15 in racing pigeons. The most prevalent STs were ST155 and ST20. ST155 exhibited the widest distribution, being abundant in both racing and meat pigeons across six provinces. ST155 was found to be identified as colistin-resistant and ESBL-producing *E. coli* in poultry in Zimbabwe [29]. Moreover, in line with Blaak et al. [30], every ST155 ESBL-EC isolates found in our investigation showed positive for *bla*_CTX-M_, whereas ST20 is the main ST type of meat pigeon source isolates in this study, which has been frequently identified as being associated with atypical enteropathogenic *E. coli* (aEPEC) [31]. It has also been stated to be linked to *mcr-1*-positive *E. coli* causing post-weaning diarrhea in Chinese pigs [32]. Notably, we found two ST131 isolates in racing pigeons. According to reports, ST131 was a high-risk clone of ESBL-EC that is widespread in both humans and animals and is thought to be the cause of worldwide epidemics of MDR and highly virulent *E. coli* infections [33]. Furthermore, we identified ST10 in the pigeons. After ST131, ST10 was reportedly the third most common clone globally among ESBL-EC strains [34]. Furthermore, among healthy individuals in France, it is one of the predominant clones [35]. These ST types are interspecies clones and have the potential for widespread distribution in both humans and animals. Overall, the findings of our investigation demonstrated that shared ST clones exist in pigeon populations, emphasizing the significance of comprehending the possibility of ESBL-EC transmission between pigeons and humans.

Genetic analysis of 95 ESBL-EC isolates revealed their distribution across seven phylogroups: A, B1, B2, C, D, E, and F. E and F phylogroups were exclusively found in meat pigeons, while the remaining five phylogenetic groups were identified in both meat pigeons and racing pigeons. Among these, isolates from racing pigeons primarily belonged to the B1 group, while isolates from meat pigeons showed a more diverse distribution, with a notable presence in B1, A, E, and D groups. It is reported six types (B2, D, B1, A, F, and *clade* I) in domestic pigeons in Slovakia, whereas racing pigeons only had three types (E, D, and B2) [20], which is similar to our findings. It is well-documented that different phylogenetic groups of *E. coli* display differential host tropisms. For instance, Johnson et al. [36] reported that the majority of human patient-derived strains of extra-intestinal pathogenic *E. coli* (ExPEC) belonged to group B2, while groups A, B1, and D (as the main types in our study) mostly belonged to avian pathogenic *E. coli* (APEC). Notably, strains of APEC were also discovered to provide potential threats to human health [37].

A total of 51 serotypes were identified, with 37 distinct serotypes in meat pigeons and 20 serotypes in racing pigeons. Moreover, there were four common serotypes between the two groups. It is evident that meat pigeons possess a higher diversity of serotypes compared to racing pigeons. O antigen is the major virulence factor of *E. coli* and correlates with its pathogenicity [38]. In the most common serotypes we predicted, O51, O8, O4, O25, O6, and O1 may possess pathogenic potential. For example, previous studies have reported that O51:H40 may cause diarrhea, O8 may produce Shiga Toxin 2 subtype, and O1, O4, and O6 are commonly isolated serotypes in uropathogenic *E. coli* (UPEC) infections [39, 40]. Notably, the O25:H4 ST131 strain is reported as a globally disseminated clone associated with high virulence and antibiotic resistance, as well as serious infections [41]. However, it is not sufficient to predict the pathogenicity of strains in pigeons solely based on serotypes. Future work should investigate the pathogenic potential of those isolates.

We observed the MDR rate of isolates was as high as 95.79%, with the potential for combined resistance to up to eight classes of antibiotics. This rate is significantly higher than 24.8% MDR rate in Qingdao pigeons and 63% MDR rate in South Brazil pigeons [16, 18]. The variation between different places and the quick growth of AMR might be a possible explanation for this disparity. As depicted in our study, there are differences in AMR between regions. Strains from Hebei, Jiangsu, and Hunan provinces exhibited higher resistance rates toward antibiotics, while Fujian province had relatively lower resistance rates. This implies that different regions may face varying pressures in terms of antibiotic usage and subsequent changes in AMR patterns. Interestingly, ESBL-EC strains isolated from racing pigeons exhibited significantly higher resistance rates toward antibiotics in compared to these isolates from meat pigeons, which is in line with the finding reported by Dobroslava et al. [20]. The variation in resistance rates may be attributed to the fact that meat pigeons, as consumption animals, would have stricter antibiotic regulations and controls. On the other hand, racing pigeons often undergo long-distance training and exposure, which may lead to physical exhaustion and an increased susceptibility to illnesses, thereby necessitating a higher frequency of antibiotic use for treatment. Such a high rate of resistance may cause a limited availability of antibiotic therapy in the clinic.

We predicted a total of 12 types of ESBL genes. Moreover, 67.37% of strains had at least two *bla*, along with four types of *bla* in five isolates. The reason for such an abundance of *bla* is likely to be related to plasmid-mediated HGT, in addition to selective pressure from antibiotics [7, 10]. Research has indicated that CTX-M lactamase is widely distributed globally, with a predominance of CTX-M-15 and CTX-M-14 in humans as well as animals [42]. Nevertheless, we found no CTX-M-15 strains in the pigeons and only nine strains of CTX-M-14. Rather, the most prevalent CTX-M that we observed were *bla*_CTX-M−55_ (*n* = 52) and *bla*_CTX-M−65_ (*n* = 20). With 99.3% amino acid similarity to CTX-M-15, CTX-M-55 is a variation that shows greater hydrolytic action against ceftazidime [43]. It was Asia's second most common *bla*, particularly in China [44]. Previous research, as well as our own, has shown that *fosA*, *mcr*, *bla*_NDM_, and *tet*(X) genes are commonly co-transmitted with *bla*_CTX-M−55_ positive *E. coli* [45]. The *bla*_CTX-M−65_, on the other hand, is frequently detected in humans, birds, and food animals [46, 47]. Additionally, studies have shown a high prevalence of ESBL-EC strains carrying *bla*_CTX-M−65_ and *bla*_CTX-M−55_ in untreated chicken meat and chickens from Vietnam and Korea, which is similar to our results [48, 49]. This strongly supports that these genes have a strong propensity for widespread dissemination among humans and animals.

We also found other ARGs besides *bla*. The most common genes were *aph*(*6*)-*Id*, *tet*(A), *sul2*, *and qnrS1*, which correspond to resistance to aminoglycosides, tetracyclines, sulfonamides, and quinolones, respectively. The resistance rates of aminoglycosides, tetracyclines, sulfonamides, and quinolones were 93.68%, 94.74%, 91.58%, and 67.37%. The positive rate of ARGs mediating them was 63.16%, 92.63%, 77.89%, and 77.89%, respectively. It should be noted, nevertheless, that phenotypic resistance does not always result from the presence of resistance genes and *vise versa* [50, 51]. This is because AMR is regulated by many factors, including known regulation of ARGs expression (i.e., resistance mutations in endogenous genes, ectopic overexpression), drug targets modification or protection, and molecular mechanisms of resistance [52]. Seven isolates with the colistin resistance gene *mcr* and one with the tigecycline resistance gene *tet*(X4) were especially concerning. Furthermore, it is well-recognized that tigecycline and colistin are the final options for treating MDR Gram-negative infections [53, 54]. Furthermore, the conjugation experiment demonstrated the effective transfer of plasmids containing *tet*(X4) and *mcr-1.1* to *E. coli* C600. It is noteworthy that these seven isolates were obtained from meat pigeons, which have direct and close contact with humans *via* the food chain. It made clear how urgently stringent monitoring and practical containment were needed to stop ARGs from spreading farther into the environment.

Of the 33 plasmid replicon types predicted in isolates, the most common were IncFIB(AP001918), Incl1-I(Alpha), IncHI2, and IncHI2A. Several studies have reported that these replicons have been found in patients, pregnant mothers, farm workers, avian, migratory birds, pigs, and sewage [12, 13, 55, 56]. Our findings align with previous associations that link them to resistance against a range of antibiotics, including *β*-lactams, polymyxins, aminoglycosides, tetracyclines, and sulfonamides [12, 55, 56]. Further evidence that plasmids may mediate the cross-species transmission of ARGs and ultimately to humans comes from reports that the same ESBL genes or plasmids have been discovered in livestock and agricultural laborers on the same farm [57].

## 5. Conclusions

To summarize, we report that the majority of ESBL-EC strains in pigeons show MDR. Notably, racing pigeons and meat pigeons exhibit distinct drug resistance patterns. Moreover, the phylogenetic groups, MLSTs, and serotypes of the isolates in meat pigeons are more diverse than those in racing pigeons. Despite some limitations, this study is the first to provide the most thorough analysis of ESBL-EC isolates in pigeons and provides the evidence that racing and meat pigeons are reservoirs of ESBL-EC isolates, having the potential to the dissemination of ARGs to humans and animals. Ongoing surveillance of MDR *E. coli* in pigeons is imperative within a One Health framework to curtail the dissemination of such pathogens.

## Figures and Tables

**Table 1:** Separation of samples from different breeds of pigeons and different cities.

Source	Region	Total samples	*E. coli* isolates (prevalence)	ESBL-EC isolates (prevalence)
Racing pigeon	Jiangsu	33	33 (100.00%)	18 (54.55%)
	Fujian	15	12 (80.00%)	6 (40.00%)
	Ningxia	19	16 (84.21%)	11 (57.89%)
	Shaanxi	13	12 (92.31%)	6 (46.15%)

Meat pigeon	Hebei	26	24 (92.31%)	22 (84.62%)
	Henan	15	11 (73.33%)	10 (66.67%)
	Hunan	26	25 (96.15%)	22 (84.62%)

Subtotal		147	133 (90.48%)	95 (64.63%)

## Data Availability

The data used to support the findings of this study are included within the article.

## Supporting Information

Supporting Information Figure S1: clustering analysis of the AMR in 95 ESBL-EC isolates. Figure S2: conjugation assay of four isolates. Table S1: PCR primers used in conjugation assay. Table S2: AMR patterns of 95 ESCL-EC isolates. Table S3: summary of detailed data involved in this study.
