# Supplementary material for: Genome-Based Molecular Diversity of Extended-Spectrum β-Lactamase-Producing Escherichia coli From Pigeons in China
Source: Transbound Emerg Dis. 2024 Oct 1;2024:1828830. doi: 10.1155/2024/1828830 (PMC12017244; doi:10.1155/2024/1828830)
Supplement: Supporting Information — Figure S1: clustering analysis of the AMR in 95 ESBL-EC isolates. Figure S2: conjugation assay of four isolates. Table S1: PCR primers used in conjugation assay. Table S2: AMR patterns of 95 ESCL-EC isolates. Table S3: summary of detailed data involved in this study. [file 1828830.f1.docx]

**
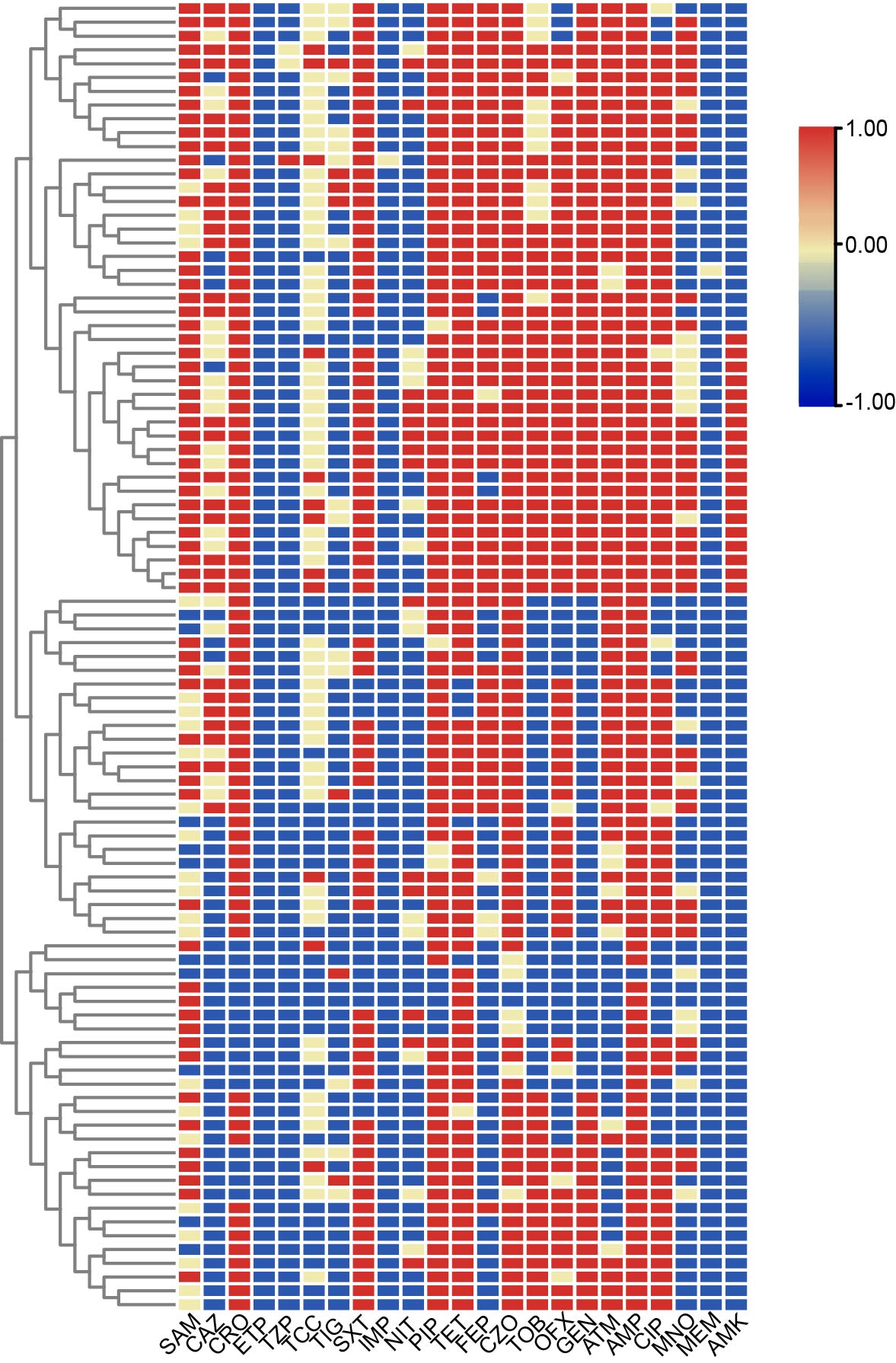
Supplementary Figure 1. Clustering analysis of the AMR in 95 ESBL-EC isolates.**

**Supplementary Figure 2. Conjugation assay of four isolates.**


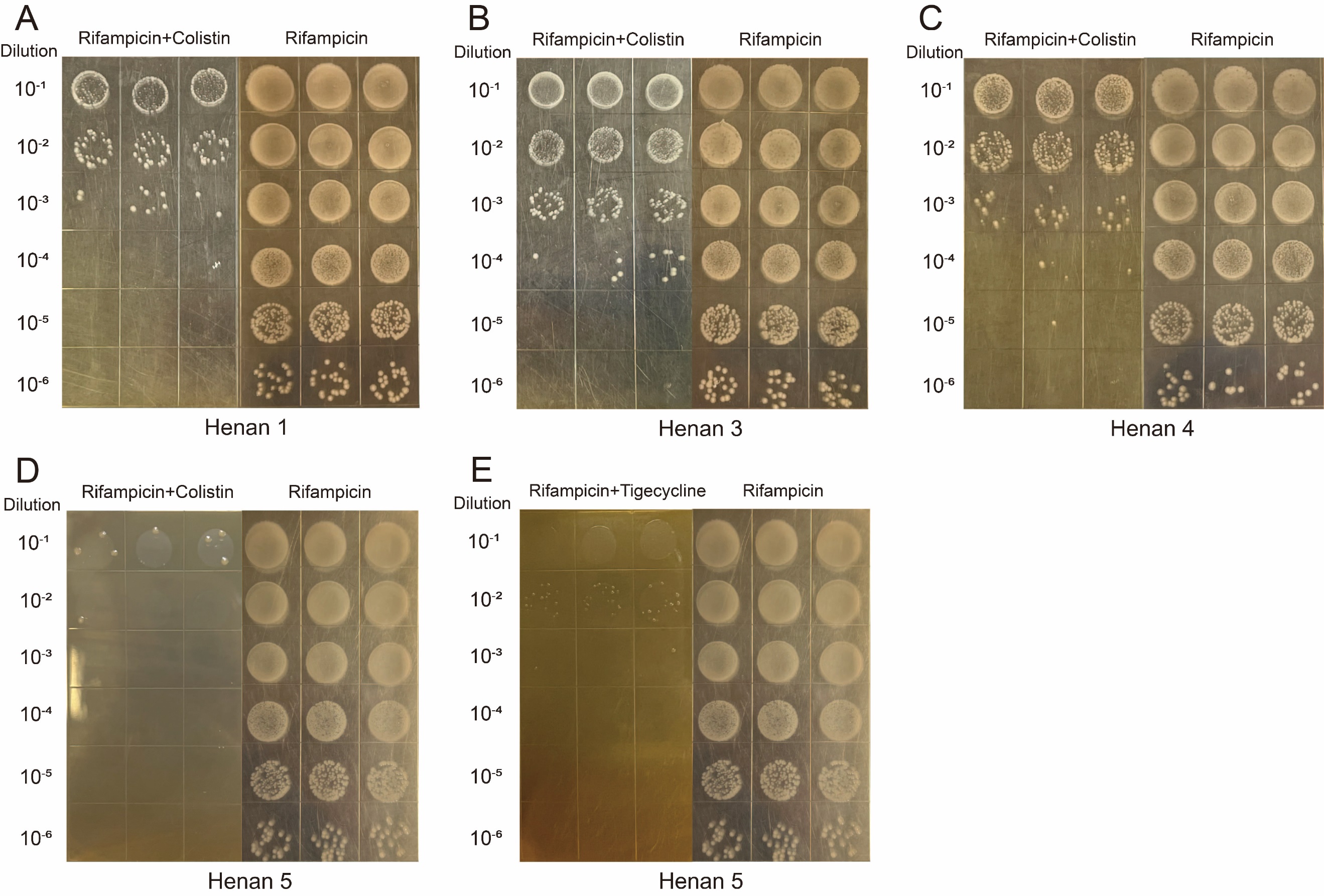


(A) Conjugation assay of Henan 1 carrying *mcr-1.1*. (B) Conjugation assay of Henan 3 carrying *mcr-1.1*. (C) Conjugation assay of Henan 4 carrying *mcr-1.1*. (D) Conjugation assay of Henan 5 carrying *mcr-1.1*. (E) Conjugation assay of Henan 5 carrying *tet*(X4).

**Supplementary Table 1.** **PCR primers used in conjugation assay.**

| **Primer**  **name** | **Target gene** | **Amplicon size** | **Primer sequences (5′ to 3′)** | **PCR condition** |
| --- | --- | --- | --- | --- |
| Mcr-1 | *mcr-1*  (KP347127.1) | 431 bp | F: TGATACGACCATGCTCCAA  R: CGACGACGAACACCACTAG | Pre-denaturation at 98°C for 2 min. Denaturation at 98°C for 10 secs, annealing at 55°C for 10 secs and extension at 72°C for 20 secs. Final extension at 72°C for 10 min. |
| Tet(X4) | *tet*(X)  (MK134376.1) | 492 bp | F: ACAAAAAATGTAAAGCCCG  R: CAGAAGAAAATCAACGACACTAT |  |

**Supplementary Table 2. AMR patterns of 95 ESCL-EC isolates.**

| Antibiotic  Classes | Antibiotics | AMR pattern | Isolate number | Percentage  % |
| --- | --- | --- | --- | --- |
| 1 | 2 | AMP-PIP | 1 | 1.05 |
| 1 | 2 | TIG-TET | 1 | 1.05 |
| 2 | 3 | AMP-SAM-TET | 2 | 2.11 |
| 3 | 4 | AMP-SAM-TET-SXT | 1 | 1.05 |
| 3 | 5 | CRO-CZO-AMP-PIP-TOB | 1 | 1.05 |
| 3 | 5 | AMP-PIP-TET-CIP-SXT | 1 | 1.05 |
| 3 | 5 | AMP-SAM-TET-SXT-NIT | 1 | 1.05 |
| 4 | 5 | CZO-AMP-PIP-TET-SXT | 1 | 1.05 |
| 4 | 6 | CRO-CZO-AMP-PIP-ATM-TET | 2 | 2.11 |
| 4 | 6 | CRO-CZO-AMP-PIP-GEN-TOB | 1 | 1.05 |
| 4 | 6 | CZO-AMP-PIP-TCC-SAM-TET | 1 | 1.05 |
| 4 | 7 | CRO-CZO-AMP-PIP-ATM-OFL-CIP | 1 | 1.05 |
| 4 | 7 | CRO-CZO-AMP-ATM-SAM-TET-SXT | 1 | 1.05 |
| 4 | 7 | CRO-CZO-AMP-TET-OFL-CIP-SXT | 2 | 2.11 |
| 4 | 8 | CRO-CZO-CZX-AMP-PIP-ATM-TET-NIT | 1 | 1.05 |
| 4 | 8 | CRO-CZO-AMP-PIP-SAM-GEN-TOB-TET | 1 | 1.05 |
| 4 | 8 | CRO-CZO-AMP-PIP-MNO-TET-OFL-CIP | 1 | 1.05 |
| 5 | 9 | CAZ-CRO-CZO-CZX-AMP-PIP-ATM-MNO-TET | 1 | 1.05 |
| 5 | 9 | CAZ-CRO-CZO-CZX-AMP-PIP-ATM-OFL-CIP | 2 | 2.11 |
| 5 | 9 | CRO-CZO-AMP-PIP-SAM-GEN-TOB-TET-SXT | 1 | 1.05 |
| 5 | 9 | CRO-CZO-AMP-PIP-ATM-SAM-MNO-TET-SXT | 1 | 1.05 |
| 5 | 9 | CRO-CZO-AMP-PIP-ATM-GEN-TOB-TET-SXT | 1 | 1.05 |
| 5 | 9 | CRO-CZO-AMP-PIP-ATM-MNO-TET-OFL-CIP | 1 | 1.05 |
| 5 | 9 | CRO-CZO-AMP-PIP-ATM-TET-OFL-CIP-SXT | 1 | 1.05 |
| 5 | 9 | CRO-CZO-AMP-PIP-TET-OFL-CIP-SXT-NIT | 1 | 1.05 |
| 5 | 9 | CZO-AMP-PIP-SAM-MNO-TET-OFL-CIP-SXT | 1 | 1.05 |
| 5 | 9 | AMP-PIP-SAM-GEN-TOB-TET-OFL-CIP-SXT | 1 | 1.05 |
| 6 | 10 | CAZ-CRO-CZO-CZX-AMP-PIP-ATM-SAM-OFL-CIP | 1 | 1.05 |
| 6 | 10 | CRO-CZO-CZX-AMP-PIP-ATM-SAM-MNO-TET-SXT | 1 | 1.05 |
| 6 | 10 | CRO-CZO-AMP-PIP-GEN-TOB-TET-OFL-CIP-SXT | 3 | 3.16 |
| 6 | 10 | CZO-AMP-PIP-SAM-GEN-TOB-TIG-TET-CIP-SXT | 1 | 1.05 |
| 6 | 10 | CZO-AMP-PIP-SAM-MNO-TET-OFL-CIP-SXT-NIT | 1 | 1.05 |
| 6 | 11 | CAZ-CRO-CZO-CZX-AMP-PIP-ATM-SAM-GEN-TET-SXT | 1 | 1.05 |
| 6 | 11 | CAZ-CRO-CZO-CZX-AMP-PIP-ATM-TET-OFL-CIP-SXT | 1 | 1.05 |
| 6 | 11 | CRO-CZO-CZX-AMP-PIP-ATM-SAM-GEN-MNO-TET-SXT | 1 | 1.05 |
| 6 | 11 | CRO-CZO-CZX-AMP-PIP-ATM-SAM-TET-OFL-CIP-SXT | 1 | 1.05 |
| 6 | 11 | CRO-CZO-CZX-AMP-PIP-ATM-MNO-TET-OFL-CIP-SXT | 1 | 1.05 |
| 6 | 11 | CRO-CZO-CZX-AMP-PIP-GEN-TOB-TET-OFL-CIP-SXT | 1 | 1.05 |
| 6 | 11 | CRO-CZO-AMP-PIP-ATM-SAM-GEN-TOB-TET-CIP-SXT | 1 | 1.05 |
| 6 | 11 | CRO-CZO-AMP-PIP-ATM-SAM-MNO-TET-OFL-CIP-SXT | 1 | 1.05 |
| 6 | 11 | CRO-CZO-AMP-PIP-ATM-TCC-TET-OFL-CIP-SXT-NIT | 1 | 1.05 |
| 6 | 11 | CRO-CZO-AMP-PIP-ATM-GEN-TOB-TET-OFL-CIP-SXT | 2 | 2.11 |
| 6 | 11 | CZO-AMP-PIP-SAM-GEN-TOB-MNO-TET-OFL-CIP-SXT | 1 | 1.05 |
| 6 | 12 | CAZ-CRO-CZO-CZX-AMP-PIP-ATM-SAM-TET-OFL-CIP-SXT | 1 | 1.05 |
| 6 | 12 | CRO-CZO-CZX-AMP-PIP-SAM-GEN-TOB-TET-OFL-CIP-SXT | 2 | 2.11 |
| 6 | 12 | CRO-CZO-CZX-AMP-PIP-ATM-SAM-TIG-MNO-TET-OFL-CIP | 1 | 1.05 |
| 6 | 12 | CRO-CZO-CZX-AMP-ATM-SAM-GEN-TOB-MNO-TET-OFL-CIP | 1 | 1.05 |
| 7 | 12 | CAZ-CRO-CZO-CZX-AMP-PIP-ATM-SAM-GEN-MNO-TET-SXT | 1 | 1.05 |
| 7 | 12 | CAZ-CRO-CZO-CZX-AMP-PIP-ATM-GEN-TET-OFL-CIP-SXT | 1 | 1.05 |
| 7 | 12 | CRO-CZO-AMP-PIP-ATM-GEN-TOB-TET-OFL-CIP-SXT-NIT | 1 | 1.05 |
| 7 | 12 | CZO-AMP-PIP-TCC-SAM-GEN-TOB-MNO-TET-OFL-CIP-SXT | 1 | 1.05 |
| 7 | 13 | CAZ-CRO-CZO-CZX-AMP-PIP-ATM-SAM-MNO-TET-OFL-CIP-SXT | 1 | 1.05 |
| 7 | 13 | CAZ-CRO-CZO-CZX-AMP-PIP-ATM-GEN-TOB-TET-OFL-CIP-SXT | 2 | 2.11 |
| 7 | 13 | CAZ-CRO-CZO-CZX-AMP-PIP-ATM-GEN-TIG-TET-OFL-CIP-SXT | 1 | 1.05 |
| 7 | 13 | CAZ-CRO-CZO-AMP-PIP-ATM-SAM-GEN-TOB-TET-OFL-CIP-SXT | 1 | 1.05 |
| 7 | 13 | CAZ-CRO-CZO-AMP-PIP-ATM-SAM-GEN-MNO-TET-OFL-CIP-SXT | 1 | 1.05 |
| 7 | 13 | CRO-CZO-CZX-AMP-PIP-ATM-SAM-GEN-TOB-AMK-TET-OFL-CIP | 1 | 1.05 |
| 7 | 13 | CRO-CZO-CZX-AMP-PIP-ATM-SAM-GEN-TOB-MNO-TET-CIP-SXT | 1 | 1.05 |
| 7 | 13 | CRO-CZO-CZX-AMP-PIP-ATM-SAM-GEN-TOB-TET-OFL-CIP-SXT | 1 | 1.05 |
| 7 | 13 | CRO-CZO-CZX-AMP-PIP-ATM-SAM-GEN-TET-OFL-CIP-SXT-NIT | 1 | 1.05 |
| 7 | 14 | CAZ-CRO-CZO-CZX-AMP-PIP-ATM-SAM-GEN-TIG-TET-OFL-CIP-SXT | 1 | 1.05 |
| 7 | 14 | CAZ-CRO-CZO-CZX-AMP-PIP-ATM-SAM-GEN-MNO-TET-OFL-CIP-SXT | 3 | 3.16 |
| 7 | 14 | CRO-CZO-CZX-AMP-PIP-ATM-SAM-GEN-TOB-AMK-TET-OFL-CIP-SXT | 2 | 2.11 |
| 7 | 14 | CRO-CZO-CZX-AMP-PIP-ATM-SAM-GEN-TOB-TIG-TET-OFL-CIP-SXT | 1 | 1.05 |
| 7 | 14 | CRO-CZO-CZX-AMP-PIP-ATM-SAM-GEN-TOB-MNO-TET-OFL-CIP-SXT | 1 | 1.05 |
| 7 | 14 | CRO-CZO-CZX-AMP-PIP-ATM-SAM-TOB-AMK-TET-OFL-CIP-SXT-NIT | 1 | 1.05 |
| 7 | 14 | CRO-CZO-CZX-AMP-PIP-ATM-TCC-SAM-GEN-TOB-AMK-TET-OFL-SXT | 1 | 1.05 |
| 7 | 14 | CRO-CZO-AMP-PIP-ATM-SAM-GEN-TOB-AMK-MNO-TET-OFL-CIP-SXT | 1 | 1.05 |
| 7 | 14 | CRO-CZO-AMP-PIP-ATM-SAM-GEN-TOB-AMK-TET-OFL-CIP-SXT-NIT | 1 | 1.05 |
| 7 | 15 | CAZ-CRO-CZO-CZX-AMP-PIP-ATM-TCC-SAM-AMK-MNO-TET-OFL-CIP-SXT | 2 | 2.11 |
| 7 | 15 | CRO-CZO-CZX-AMP-PIP-ATM-SAM-GEN-TOB-AMK-MNO-TET-OFL-CIP-SXT | 2 | 2.11 |
| 7 | 15 | CRO-CZO-CZX-AMP-PIP-ATM-PTCC-SAM-GEN-TOB-TET-OFL-CIP-SXT | 1 | 1.05 |
| 7 | 15 | CRO-CZO-CZX-AMP-PIP-ATM-TCC-SAM-GEN-TOB-MNO-TET-OFL-CIP-SXT | 1 | 1.05 |
| 8 | 16 | CAZ-CRO-CZO-CZX-AMP-PIP-ATM-SAM-GEN-TOB-AMK-MNO-TET-OFL-CIP-SXT | 1 | 1.05 |
| 8 | 16 | CAZ-CRO-CZO-CZX-AMP-PIP-ATM-TCC-SAM-GEN-TOB-AMK-TET-OFL-CIP-SXT | 1 | 1.05 |
| 8 | 16 | CAZ-CRO-CZO-AMP-PIP-ATM-TCC-SAM-GEN-TOB-AMK-MNO-TET-OFL-CIP-SXT | 1 | 1.05 |
| 8 | 16 | CRO-CZO-CZX-AMP-PIP-ATM-SAM-GEN-TOB-AMK-MNO-TET-OFL-CIP-SXT-NIT | 2 | 2.11 |
| 8 | 17 | CAZ-CRO-CZO-CZX-AMP-PIP-ATM-SAM-GEN-TOB-AMK-MNO-TET-OFL-CIP-SXT-NIT | 2 | 2.11 |
| 8 | 17 | CAZ-CRO-CZO-CZX-AMP-PIP-ATM-TCC-SAM-GEN-TOB-AMK-MNO-TET-OFL-CIP-SXT | 1 | 1.05 |

| Strain | Species | Provience | City | Breed | MLST | FumC | FimH | Serotype | Phylogenetic groups |
| --- | --- | --- | --- | --- | --- | --- | --- | --- | --- |
| HuNan1 | *Escherichia coli* | Hunan | Changde | Meat pigeon | 132 | 50 | 299 | O3:H1 | D |
| HuNan2 | *Escherichia coli* | Hunan | Changde | Meat pigeon | 7435 | 29 | 86 | O6:H28 | B1 |
| HuNan3 | *Escherichia coli* | Hunan | Changde | Meat pigeon | 1485 | 231 | 58 | O1:H34 | F |
| HuNan4 | *Escherichia coli* | Hunan | Changde | Meat pigeon | 155 | 4 | 32 | O9:H51 | B1 |
| HuNan5 | *Escherichia coli* | Hunan | Changde | Meat pigeon | 3460 | 23 | 32 | O51:H28 | B1 |
| HuNan6 | *Escherichia coli* | Hunan | Changde | Meat pigeon | 155 | 4 | 32 | O51:H4 | B1 |
| HuNan7 | *Escherichia coli* | Hunan | Changde | Meat pigeon | 6164 | 43 | 225 | O175:H5 | B2 |
| HuNan8 | *Escherichia coli* | Hunan | Changde | Meat pigeon | 155 | 4 | 32 | O51:H4 | B1 |
| HuNan9 | *Escherichia coli* | Hunan | Changde | Meat pigeon | 602 | 19 | 86 | H21 | B1 |
| HuNan10 | *Escherichia coli* | Hunan | Changde | Meat pigeon | 7435 | 29 | 86 | O6:H28 | B1 |
| HuNan11 | *Escherichia coli* | Hunan | Changde | Meat pigeon | 132 | 50 | 299 | O3:H1 | D |
| HuNan12 | *Escherichia coli* | Hunan | Changde | Meat pigeon | 10 | 11 | 54 | O16:H48 | A |
| HuNan13 | *Escherichia coli* | Hunan | Changde | Meat pigeon | 1485 | 231 | 58 | O1:H34 | F |
| HuNan14 | *Escherichia coli* | Hunan | Changde | Meat pigeon | 7435 | 29 | 86 | O6:H28 | B1 |
| HuNan15 | *Escherichia coli* | Hunan | Changde | Meat pigeon | 155 | 4 | 32 | O51:H4 | B1 |
| HuNan16 | *Escherichia coli* | Hunan | Changde | Meat pigeon | 2847 | 266 | 1310 | O53:H9 | E |
| HuNan17 | *Escherichia coli* | Hunan | Changde | Meat pigeon | 132 | 50 | 299 | O3:H1 | D |
| HuNan18 | *Escherichia coli* | Hunan | Changde | Meat pigeon | 7435 | 29 | 86 | O6:H28 | B1 |
| HuNan19 | *Escherichia coli* | Hunan | Changde | Meat pigeon | 58 | 4 | 86 | O103:H16 | B1 |
| HuNan20 | *Escherichia coli* | Hunan | Changde | Meat pigeon | 1485 | 231 | 58 | O1:H34 | F |
| HuNan21 | *Escherichia coli* | Hunan | Changde | Meat pigeon | 3576 | 29 | 68 | O8:H7 | B1 |
| HuNan22 | *Escherichia coli* | Hunan | Changde | Meat pigeon | 2175 | 4 | 1325 | O86:H30 | B1 |
| HeNan1 | *Escherichia coli* | Henan | Pingdingshan | Meat pigeon | 38 | 26 | 65 | O1:H15 | D |
| HeNan2 | *Escherichia coli* | Henan | Pingdingshan | Meat pigeon | 7427 | 331 | 986 | H6 | D |
| HeNan3 | *Escherichia coli* | Henan | Pingdingshan | Meat pigeon | 1011 | 4 | 31 | O51:H45 | E |
| HeNan4 | *Escherichia coli* | Henan | Pingdingshan | Meat pigeon | 1148 | 95 | 32 | O163:H7 | B1 |
| HeNan5 | *Escherichia coli* | Henan | Pingdingshan | Meat pigeon | 156 | 29 | 38 | O51:H28 | B1 |
| HeNan6 | *Escherichia coli* | Henan | Pingdingshan | Meat pigeon | 2973 | 95 | 31 | H16 | A |
| HeNan7 | *Escherichia coli* | Henan | Pingdingshan | Meat pigeon | 2008 | 6 | 31 | H2 | B1 |
| HeNan8 | *Escherichia coli* | Henan | Pingdingshan | Meat pigeon | 155 | 4 | 32 | O25:H51 | B1 |
| HeNan9 | *Escherichia coli* | Henan | Pingdingshan | Meat pigeon | 155 | 4 | 32 | O25:H51 | B1 |
| HeNan10 | *Escherichia coli* | Henan | Pingdingshan | Meat pigeon | 155 | 4 | 32 | O25:H51 | B1 |
| HeBei1 | *Escherichia coli* | Hebei | Shijiazhuang | Meat pigeon | 1196 | 6 | 31 | O29:H8 | B1 |
| HeBei2 | *Escherichia coli* | Hebei | Shijiazhuang | Meat pigeon | 1196 | 6 | 31 | O29:H8 | B1 |
| HeBei3 | *Escherichia coli* | Hebei | Shijiazhuang | Meat pigeon | 1196 | 6 | 31 | O29:H8 | B1 |
| HeBei4 | *Escherichia coli* | Hebei | Shijiazhuang | Meat pigeon | 1196 | 6 | 31 | O29:H8 | B1 |
| HeBei5 | *Escherichia coli* | Hebei | Shijiazhuang | Meat pigeon | 2107 | 65 | 32 | O8:H19 | B1 |
| HeBei6 | *Escherichia coli* | Hebei | Hengshui | Meat pigeon | 1011 | 4 | 31 | O54:H21 | E |
| HeBei7 | *Escherichia coli* | Hebei | Hengshui | Meat pigeon | 1011 | 4 | 31 | O54:H21 | E |
| HeBei8 | *Escherichia coli* | Hebei | Hengshui | Meat pigeon | 155 | 4 | 32 | O68:H9 | B1 |
| HeBei9 | *Escherichia coli* | Hebei | Hengshui | Meat pigeon | 196 | 19 | 87 | O8:H7 | E |
| HeBei10 | *Escherichia coli* | Hebei | Hengshui | Meat pigeon | 1196 | 6 | 31 | H28 | B1 |
| HeBei11 | *Escherichia coli* | Hebei | Cangzhou | Meat pigeon | 1011 | 4 | 31 | O168:H28 | E |
| HeBei12 | *Escherichia coli* | Hebei | Cangzhou | Meat pigeon | 2473 | 19 | 38 | O168 | B1 |
| HeBei13 | *Escherichia coli* | Hebei | Cangzhou | Meat pigeon | 6335 | 27 | 23 | O51:H26 | C |
| HeBei14 | *Escherichia coli* | Hebei | Cangzhou | Meat pigeon | 1011 | 4 | 331 | O32:H45 | E |
| HeBei15 | *Escherichia coli* | Hebei | Cangzhou | Meat pigeon | 1011 | 4 | 331 | O32:H45 | E |

**Supplementary Table 3. Summary of detailed data involved in this study.**

| Strain | Species | Provience | City | Breed | MLST | FumC | FimH | Serotype | Phylogenetic groups |
| --- | --- | --- | --- | --- | --- | --- | --- | --- | --- |
| HeBei16 | *Escherichia coli* | Hebei | Cangzhou | Meat pigeon | 646 | 96 | 5 | O166:H6 | B2 |
| HeBei17 | *Escherichia coli* | Hebei | Cangzhou | Meat pigeon | 224 | 4 | 61 | O181:H23 | B1 |
| HeBei18 | *Escherichia coli* | Hebei | Cangzhou | Meat pigeon | 162 | 65 | 32 | O78:H23 | B1 |
| HeBei19 | *Escherichia coli* | Hebei | Tangshan | Meat pigeon | 1086 | 4 | 32 | O8:H14 | B1 |
| HeBei20 | *Escherichia coli* | Hebei | Tangshan | Meat pigeon | 20 | 4 | 25 | O4:H2 | B1 |
| HeBei21 | *Escherichia coli* | Hebei | Tangshan | Meat pigeon | 7058 | 23 | 40 | O148:H30 | A |
| HeBei22 | *Escherichia coli* | Hebei | Tangshan | Meat pigeon | 1589 | 4 | 65 | O45:H7 | B1 |
| JiangSu1 | *Escherichia coli* | Jiangsu | Yancheng | Racing pigeon | 1423 | 4 | 38 | O140:H21 | B1 |
| JiangSu2 | *Escherichia coli* | Jiangsu | Yancheng | Racing pigeon | 1423 | 4 | 38 | O140:H21 | B1 |
| JiangSu3 | *Escherichia coli* | Jiangsu | Yancheng | Racing pigeon | 155 | 4 | 32 | O51:H51 | B1 |
| JiangSu4 | *Escherichia coli* | Jiangsu | Yancheng | Racing pigeon | 155 | 4 | 32 | O51:H51 | B1 |
| JiangSu5 | *Escherichia coli* | Jiangsu | Yancheng | Racing pigeon | 20 | 4 | 25 | O4:H2 | B1 |
| JiangSu6 | *Escherichia coli* | Jiangsu | Yancheng | Racing pigeon | 20 | 4 | 25 | O4:H2 | B1 |
| JiangSu7 | *Escherichia coli* | Jiangsu | Yancheng | Racing pigeon | 20 | 4 | 25 | O4:H2 | B1 |
| JiangSu8 | *Escherichia coli* | Jiangsu | Yancheng | Racing pigeon | 1423 | 4 | 38 | O140:H21 | B1 |
| JiangSu9 | *Escherichia coli* | Jiangsu | Yancheng | Racing pigeon | 38 | 26 | 65 | O1:H15 | D |
| JiangSu10 | *Escherichia coli* | Jiangsu | Yancheng | Racing pigeon | 20 | 4 | 25 | O4:H2 | B1 |
| JiangSu11 | *Escherichia coli* | Jiangsu | Yancheng | Racing pigeon | 46 | 7 | 34 | O9a:H4 | A |
| JiangSu12 | *Escherichia coli* | Jiangsu | Yancheng | Racing pigeon | 46 | 7 | 34 | O9a:H4 | A |
| JiangSu13 | *Escherichia coli* | Jiangsu | Yancheng | Racing pigeon | 155 | 4 | 32 | O160:H51 | B1 |
| JiangSu14 | *Escherichia coli* | Jiangsu | Yancheng | Racing pigeon | 90 | 4 | 142 | O8:H9 | C |
| JiangSu15 | *Escherichia coli* | Jiangsu | Yancheng | Racing pigeon | 6756 | 7 | 23 | O159:H45 | A |
| JiangSu16 | *Escherichia coli* | Jiangsu | Yancheng | Racing pigeon | 46 | 7 | 34 | O9a:H4 | A |
| JiangSu17 | *Escherichia coli* | Jiangsu | Yancheng | Racing pigeon | 90 | 4 | 142 | O8:H9 | C |
| JiangSu18 | *Escherichia coli* | Jiangsu | Yancheng | Racing pigeon | 46 | 7 | 34 | O9a:H4 | A |
| FuJian2 | *Escherichia coli* | Fujian | Fuzhou | Racing pigeon | 131 | 40 | 22 | O25:H4 | B2 |
| FuJian3 | *Escherichia coli* | Fujian | Fuzhou | Racing pigeon | 1086 | 4 | 32 | O8:H14 | B1 |
| FuJian4 | *Escherichia coli* | Fujian | Fuzhou | Racing pigeon | 131 | 40 | 22 | O25:H4 | B2 |
| FuJian5 | *Escherichia coli* | Fujian | Fuzhou | Racing pigeon | 20 | 4 | 25 | O45:H2 | B1 |
| FuJian6 | *Escherichia coli* | Fujian | Fuzhou | Racing pigeon | 20 | 4 | 25 | O45:H2 | B1 |
| FuJian7 | *Escherichia coli* | Fujian | Fuzhou | Racing pigeon | 2685 | 4 | 25 | H2 | B1 |
| NingXia1 | *Escherichia coli* | Ningxia | Wuzhong | Racing pigeon | 155 | 4 | 32 | O86:H51 | B1 |
| NingXia2 | *Escherichia coli* | Ningxia | Wuzhong | Racing pigeon | 155 | 4 | 32 | O86:H51 | B1 |
| NingXia3 | *Escherichia coli* | Ningxia | Wuzhong | Racing pigeon | 155 | 4 | 32 | O86:H51 | B1 |
| NingXia4 | *Escherichia coli* | Ningxia | Wuzhong | Racing pigeon | 155 | 4 | 32 | O86:H51 | B1 |
| NingXia5 | *Escherichia coli* | Ningxia | Wuzhong | Racing pigeon | 1706 | 29 | 38 | O116:H8 | B1 |
| NingXia6 | *Escherichia coli* | Ningxia | Wuzhong | Racing pigeon | 1706 | 29 | 38 | O116:H8 | B1 |
| NingXia7 | *Escherichia coli* | Ningxia | Wuzhong | Racing pigeon | 1706 | 29 | 38 | O116:H8 | B1 |
| NingXia8 | *Escherichia coli* | Ningxia | Wuzhong | Racing pigeon | 20 | 4 | 25 | O4:H2 | B1 |
| NingXia9 | *Escherichia coli* | Ningxia | Wuzhong | Racing pigeon | 1706 | 29 | 38 | O116:H8 | B1 |
| NingXia10 | *Escherichia coli* | Ningxia | Wuzhong | Racing pigeon | 155 | 4 | 32 | O9:H51 | B1 |
| NingXia11 | *Escherichia coli* | Ningxia | Wuzhong | Racing pigeon | 155 | 4 | 32 | O9:H51 | B1 |
| ShaanXi1 | *Escherichia coli* | Shaanxi | Xi'an | Racing pigeon | 362 | 100 | 96 | O7:H6 | D |
| ShaanXi2 | *Escherichia coli* | Shaanxi | Xi'an | Racing pigeon | 4038 | 19 | 38 | O93:H28 | B1 |
| ShaanXi3 | *Escherichia coli* | Shaanxi | Xi'an | Racing pigeon | 1798 | 4 | 39 | O8:H2 | B1 |
| ShaanXi4 | *Escherichia coli* | Shaanxi | Baoji | Racing pigeon | 155 | 4 | 32 | O185:H51 | B1 |
| ShaanXi5 | *Escherichia coli* | Shaanxi | Baoji | Racing pigeon | 224 | 4 | 61 | O21:H28 | B1 |
| ShaanXi6 | *Escherichia coli* | Shaanxi | Baoji | Racing pigeon | 224 | 4 | 61 | O21:H28 | B1 |

| Strain | Phenotypic AMR | | | | | | | | | | | |
| --- | --- | --- | --- | --- | --- | --- | --- | --- | --- | --- | --- | --- |
|  | **Cephalosporins** | | | | **Carbapenems** | | | **Penicillins** | | | | |
|  | **CAZ** | **CRO** | **CZO** | **FEP** | **MEM** | **ETP** | **IMP** | **AMP** | **PIP** | **SAM** | **TZP** | **TCC** |
| HuNan1 | <1 | 16 | >16 | <4 | <0.5 | <0.25 | <0.5 | >32 | >128 | 16/8 | <8/4 | <16/2 |
| HuNan2 | <1 | >32 | >16 | <4 | <0.5 | <0.25 | <0.5 | >32 | >128 | >32/16 | <8/4 | 64/2 |
| HuNan3 | <1 | >32 | >16 | <4 | <0.5 | <0.25 | <0.5 | >32 | >128 | 16/8 | <8/4 | 16/2 |
| HuNan4 | 4 | >32 | >16 | 8 | <0.5 | <0.25 | <0.5 | >32 | >128 | <8/4 | <8/4 | 16/2 |
| HuNan5 | <1 | >32 | >16 | <4 | <0.5 | <0.25 | <0.5 | >32 | 64 | <8/4 | <8/4 | <8/2 |
| HuNan6 | 2 | >32 | >16 | <4 | <0.5 | <0.25 | <0.5 | >32 | >128 | 16/8 | <8/4 | <8/2 |
| HuNan7 | <1 | <0.5 | 16 | <4 | <0.5 | <0.25 | <0.5 | >32 | >128 | >32/16 | <16/4 | >128/2 |
| HuNan8 | 2 | >32 | >16 | 16 | <1 | <0.25 | <0.5 | >32 | >128 | 16/8 | <8/4 | >128/2 |
| HuNan9 | <1 | <0.5 | 8 | <4 | <0.5 | <0.25 | <0.5 | >32 | >128 | >32/16 | <8/4 | 32/2 |
| HuNan10 | <1 | <0.5 | 4 | <4 | <0.5 | <0.25 | <0.5 | >32 | <16 | >32/16 | <8/4 | 16/2 |
| HuNan11 | <1 | >32 | >16 | 8 | <0.5 | <0.25 | <0.5 | >32 | >128 | >32/16 | <8/4 | 32/2 |
| HuNan12 | <1 | >32 | >16 | <4 | <0.5 | <0.25 | <0.5 | >32 | >128 | <8/4 | <8/4 | 16/2 |
| HuNan13 | <1 | >32 | >16 | <4 | <0.5 | <0.25 | <0.5 | >32 | >128 | 16/8 | <8/4 | 16/2 |
| HuNan14 | <1 | <0.5 | 4 | <4 | <0.5 | <0.25 | <0.5 | <8 | <16 | <8/4 | <8/4 | 8/2 |
| HuNan15 | <1 | >32 | >16 | <4 | <0.5 | <0.25 | <0.5 | >32 | 64 | <8/4 | <8/4 | 8/2 |
| HuNan16 | <1 | 16 | >16 | <4 | <0.5 | <0.25 | <0.5 | >32 | >128 | <8/4 | <8/4 | 16/2 |
| HuNan17 | <1 | >32 | >16 | <4 | <0.5 | <0.25 | <0.5 | >32 | >128 | 16/8 | <8/4 | 32/2 |
| HuNan18 | <1 | <0.5 | 4 | <4 | <0.5 | <0.25 | <0.5 | >32 | <16 | >32/16 | <8/4 | 8/2 |
| HuNan19 | <1 | <0.5 | 2 | <4 | <0.5 | <0.25 | <0.5 | >32 | <16 | >32/16 | <16/4 | <16/2 |
| HuNan20 | <1 | <0.5 | 4 | <4 | <0.5 | <0.25 | <0.5 | >32 | >128 | <8/4 | <8/4 | 16/2 |
| HuNan21 | <1 | >32 | >16 | <4 | <0.5 | <0.25 | <0.5 | >32 | >128 | 16/8 | <8/4 | 16/2 |
| HuNan22 | <1 | <0.5 | 4 | <4 | <0.5 | <0.25 | <0.5 | >32 | >128 | <8/4 | <8/4 | 16/2 |
| HeNan1 | >16 | >32 | >16 | >32 | <0.5 | <0.25 | <0.5 | >32 | >128 | 16/8 | <8/4 | 64/2 |
| HeNan2 | <1 | <0.5 | 8 | <4 | <0.5 | <0.25 | <0.5 | >32 | >128 | 16/8 | <8/4 | 16/2 |
| HeNan3 | 16 | >32 | >16 | >32 | <0.5 | <0.25 | <0.5 | >32 | >128 | >32/16 | <8/4 | 64/2 |
| HeNan4 | >16 | >4 | >16 | >32 | <0.5 | <0.25 | <0.5 | >32 | >128 | 16/8 | <8/4 | 32/2 |
| HeNan5 | 8 | >32 | 16 | >32 | <0.5 | <0.25 | <0.5 | >32 | >128 | >32/16 | <8/4 | 64/2 |
| HeNan6 | 8 | >32 | >16 | >32 | <0.5 | <0.25 | <0.5 | >32 | 64 | >32/16 | <8/4 | 32/2 |
| HeNan7 | 16 | >32 | >16 | >32 | <0.5 | <0.25 | <0.5 | >32 | >128 | 16/8 | <8/4 | 16/2 |
| HeNan8 | 4 | >32 | >16 | 16 | <0.5 | <0.25 | <0.5 | >32 | >128 | 16/8 | <8/4 | 32/2 |
| HeNan9 | 4 | >32 | >16 | <4 | <0.5 | <0.25 | <0.5 | >32 | >128 | 16/8 | <8/4 | 32/2 |
| HeNan10 | 2 | >32 | >16 | 16 | <0.5 | <0.25 | <0.5 | >32 | >128 | 16/8 | <8/4 | 16/2 |
| HeBei1 | >16 | >32 | >16 | 32 | <0.5 | <0.25 | <0.5 | >32 | >128 | 16/8 | <8/4 | 32/2 |
| HeBei2 | >16 | >32 | >16 | >32 | <0.5 | <0.25 | <0.5 | >32 | >128 | 16/8 | <8/4 | 32/2 |
| HeBei3 | 16 | >32 | >16 | 32 | <0.5 | <0.25 | <0.5 | >32 | >128 | 16/8 | <8/4 | 32/2 |
| HeBei4 | 16 | >32 | >16 | 32 | <0.5 | <0.25 | <0.5 | >32 | >128 | 16/8 | <8/4 | 64/2 |
| HeBei5 | <1 | >32 | >16 | >32 | 2 | <0.25 | <0.5 | >32 | >128 | >32/16 | <8/4 | 64/2 |
| HeBei6 | <1 | >32 | >8 | 8 | <0.5 | <0.25 | <0.5 | >32 | >128 | 16/8 | <8/4 | <16/2 |
| HeBei7 | <1 | >32 | >16 | >32 | <0.5 | <0.25 | <0.5 | >32 | >128 | 16/8 | <8/4 | <16/2 |
| HeBei8 | 2 | >32 | >16 | <4 | <0.5 | <0.25 | <0.5 | >32 | >128 | 16/8 | <8/4 | <16/2 |
| HeBei9 | <1 | <0.5 | 2 | <4 | <0.5 | <0.25 | <0.5 | >32 | <16 | >32/16 | <8/4 | <16/2 |
| HeBei10 | <1 | >32 | >16 | >32 | <0.5 | <0.25 | <0.5 | >32 | >128 | >32/16 | <8/4 | 64/2 |
| HeBei11 | >16 | >32 | >16 | >32 | <0.5 | <0.25 | <1 | >32 | >128 | >32/16 | 32/4 | >128/2 |
| HeBei12 | 2 | >32 | >16 | >32 | <0.5 | <0.25 | <0.5 | >32 | >128 | >32/16 | <16/4 | 64/2 |
| HeBei13 | 16 | >32 | >16 | 32 | <0.5 | <0.25 | <0.5 | >32 | >128 | >32/16 | <8/4 | 64/2 |
| HeBei14 | 8 | >32 | >16 | 32 | <0.5 | <0.25 | <0.5 | >32 | >128 | >32/16 | <8/4 | 64/2 |
| HeBei15 | 16 | >32 | >16 | >32 | <0.5 | <0.25 | <0.5 | >32 | >128 | 16/8 | <8/4 | 64/2 |

| Strain | Phenotypic AMR | | | | | | | | | | | |
| --- | --- | --- | --- | --- | --- | --- | --- | --- | --- | --- | --- | --- |
|  | **Cephalosporins** | | | | **Carbapenems** | | | **Penicillins** | | | | |
|  | **CAZ** | **CRO** | **CZO** | **FEP** | **MEM** | **ETP** | **IMP** | **AMP** | **PIP** | **SAM** | **TZP** | **TCC** |
| HeBei16 | 8 | >32 | >16 | 32 | <0.5 | <0.25 | <0.5 | >32 | >128 | 16/8 | <8/4 | 16/2 |
| HeBei17 | >16 | >32 | >16 | >32 | <0.5 | <0.25 | <0.5 | >32 | >128 | >32/16 | <8/4 | 64/2 |
| HeBei18 | 8 | >32 | >16 | 32 | <0.5 | <0.25 | <0.5 | >32 | >128 | 16/8 | <8/4 | 16/2 |
| HeBei19 | >16 | >32 | >16 | >32 | <0.5 | <0.25 | <0.5 | >32 | >128 | >32/16 | <16/4 | 64/2 |
| HeBei20 | 16 | >32 | >16 | >32 | <0.5 | <0.25 | <0.5 | >32 | >128 | >32/16 | <8/4 | >128/2 |
| HeBei21 | 2 | >32 | >16 | <4 | <0.5 | <0.25 | <0.5 | >32 | >128 | >32/16 | <8/4 | 32/2 |
| HeBei22 | 4 | >32 | >16 | 32 | <0.5 | <0.25 | 2 | >32 | >128 | >32/16 | 128/4 | 64/2 |
| JiangSu1 | 16 | >32 | >16 | >32 | <0.5 | <0.25 | <0.5 | >32 | >128 | >32/16 | <8/4 | 32/2 |
| JiangSu2 | >16 | >32 | >16 | >32 | <0.5 | <0.25 | <0.5 | >32 | >128 | >32/16 | <8/4 | 32/2 |
| JiangSu3 | <1 | <0.5 | 16 | <4 | <0.5 | <0.25 | <0.5 | >32 | >128 | >32/16 | <8/4 | 64/2 |
| JiangSu4 | <1 | <0.5 | >16 | <4 | <0.5 | <0.25 | <0.5 | >32 | >128 | >32/16 | <16/4 | >128/2 |
| JiangSu5 | 16 | >32 | >16 | 32 | <0.5 | <0.25 | <0.5 | >32 | >128 | >32/16 | <8/4 | 64/2 |
| JiangSu6 | 16 | >32 | >16 | 32 | <0.5 | <0.25 | <0.5 | >32 | >128 | >32/16 | <8/4 | 64/2 |
| JiangSu7 | 8 | >32 | >16 | >32 | <0.5 | <0.5 | <1 | >32 | >128 | >32/16 | <8/4 | 64/2 |
| JiangSu8 | 16 | >32 | >16 | >32 | <0.5 | <0.25 | <0.5 | >32 | >128 | >32/16 | <8/4 | 64/2 |
| JiangSu9 | 8 | >32 | >16 | >32 | <0.5 | <0.25 | <0.5 | >32 | >128 | >32/16 | <8/4 | 32/2 |
| JiangSu10 | 16 | >32 | >16 | 8 | <0.5 | <0.25 | <0.5 | >32 | >128 | >32/16 | <8/4 | 64/2 |
| JiangSu11 | >16 | >32 | >16 | >32 | <0.5 | <0.25 | <0.5 | >32 | >128 | >32/16 | <8/4 | >128/2 |
| JiangSu12 | 16 | >32 | >16 | >32 | <0.5 | <0.25 | <0.5 | >32 | >128 | >32/16 | <8/4 | >128/2 |
| JiangSu13 | >16 | >32 | >16 | 8 | <0.5 | <0.25 | <0.5 | >32 | >128 | >32/16 | <8/4 | 32/2 |
| JiangSu14 | >16 | >32 | >16 | >32 | <0.5 | <0.25 | <0.5 | >32 | >128 | >32/16 | <8/4 | 64/2 |
| JiangSu15 | <1 | >32 | >16 | >32 | <0.5 | <0.25 | <0.5 | >32 | >128 | >32/16 | <8/4 | <16/2 |
| JiangSu16 | 16 | >32 | >16 | 8 | <0.5 | <0.25 | <0.5 | >32 | >128 | >32/16 | <16/4 | >128/2 |
| JiangSu17 | 8 | >32 | >16 | >32 | <0.5 | <0.25 | <0.5 | >32 | >128 | >32/16 | <8/4 | 64/2 |
| JiangSu18 | 8 | >32 | >16 | >32 | <0.5 | <0.25 | <0.5 | >32 | >128 | >32/16 | <8/4 | 64/2 |
| FuJian2 | <1 | <0.5 | 8 | <4 | <0.5 | <0.25 | <0.5 | >32 | >128 | >32/16 | <8/4 | 32/2 |
| FuJian3 | <1 | >32 | >16 | <4 | <0.5 | <0.25 | <0.5 | >32 | >128 | >32/16 | <8/4 | 32/2 |
| FuJian4 | <1 | <0.5 | 8 | <4 | <0.5 | <0.25 | <0.5 | >32 | >128 | >32/16 | <8/4 | 32/2 |
| FuJian5 | 4 | >32 | >16 | <4 | <0.5 | <0.25 | <0.5 | >32 | >128 | <8/4 | <8/4 | 8/2 |
| FuJian6 | 8 | >32 | >16 | 8 | <0.5 | <0.25 | <0.5 | >32 | >128 | <8/4 | <8/4 | 16/2 |
| FuJian7 | <1 | >32 | >16 | <4 | <1 | <0.25 | <0.5 | >32 | 64 | >32/16 | <8/4 | 32/2 |
| NingXia1 | 16 | >32 | >16 | >32 | <0.5 | <0.25 | <0.5 | >32 | >128 | >32/16 | <8/4 | 32/2 |
| NingXia2 | 8 | >32 | >16 | >32 | <0.5 | <0.25 | <0.5 | >32 | >128 | >32/16 | <8/4 | 32/2 |
| NingXia3 | 8 | >32 | >16 | 32 | <0.5 | <0.25 | <0.5 | >32 | >128 | >32/16 | <8/4 | 32/2 |
| NingXia4 | 16 | >32 | >16 | 32 | <0.5 | <0.25 | <0.5 | >32 | >128 | >32/16 | <8/4 | 32/2 |
| NingXia5 | 8 | >32 | >16 | 16 | <0.5 | <0.25 | <0.5 | >32 | >128 | >32/16 | <8/4 | 64/2 |
| NingXia6 | 4 | >32 | >16 | >32 | <0.5 | <0.25 | <0.5 | >32 | >128 | >32/16 | <8/4 | 32/2 |
| NingXia7 | 8 | >32 | >16 | >32 | <0.5 | <0.25 | <0.5 | >32 | >128 | >32/16 | <8/4 | >128/2 |
| NingXia8 | 8 | >32 | >16 | >32 | <0.5 | <0.25 | <0.5 | >32 | >128 | >32/16 | <8/4 | <16/2 |
| NingXia9 | 8 | >32 | >16 | >32 | <0.5 | <0.25 | <0.5 | >32 | >128 | >32/16 | <8/4 | 64/2 |
| NingXia10 | 8 | >32 | >16 | 32 | <0.5 | <0.25 | <0.5 | >32 | >128 | >32/16 | <8/4 | 32/2 |
| NingXia11 | 8 | >32 | >16 | >32 | <0.5 | <0.25 | <0.5 | >32 | >128 | >32/16 | <8/4 | 64/2 |
| ShaanXi1 | 8 | >32 | >16 | 32 | <0.5 | <0.25 | <0.5 | >32 | >128 | >32/16 | <8/4 | 32/2 |
| ShaanXi2 | <1 | <0.5 | 4 | <4 | <0.5 | <0.25 | <0.5 | >32 | >128 | >32/16 | <8/4 | 32/2 |
| ShaanXi3 | 8 | >32 | >16 | >32 | <0.5 | <0.25 | <0.5 | >32 | >128 | >32/16 | <8/4 | 32/2 |
| ShaanXi4 | 4 | >32 | >16 | 8 | <0.5 | <0.25 | <0.5 | >32 | >128 | >32/16 | <8/4 | 32/2 |
| ShaanXi5 | 8 | >32 | >16 | 8 | <0.5 | <0.25 | <0.5 | >32 | >128 | >32/16 | <8/4 | 64/2 |
| ShaanXi6 | 16 | >32 | >16 | >32 | <0.5 | <0.25 | <0.5 | >32 | >128 | >32/16 | <8/4 | 64/2 |

| Strain | Phenotypic AMR | | | | | | | | | | | | |
| --- | --- | --- | --- | --- | --- | --- | --- | --- | --- | --- | --- | --- | --- |
|  | **Monobactams** | **Aminoglycosides** | | | **Tetracyclines** | | | **Quinoloones** | | **Sulfanilamides** | | **Nitrofuran** | |
|  | **ATM** | **GEN** | **TOB** | **AMK** | **TIG** | **MNO** | **TET** | **OFX** | **CIP** | **SXT** | | **NIT** |  |
| HuNan1 | <1 | 8 | >16 | <16 | <1 | 4 | 8 | <1 | <1 | <2/38 | <32 | |  |
| HuNan2 | 8 | >16 | >16 | <16 | <1 | 2 | >16 | <1 | <1 | >4/76 | <32 | |  |
| HuNan3 | >16 | >16 | >16 | <16 | <1 | <1 | >16 | >8 | >4 | >4/76 | <32 | |  |
| HuNan4 | >16 | <4 | <4 | <16 | <1 | 2 | <4 | >8 | >4 | <2/38 | <32 | |  |
| HuNan5 | 8 | <4 | <4 | <16 | <1 | <1 | >16 | 8 | >4 | >4/76 | <32 | |  |
| HuNan6 | 16 | <4 | <4 | <16 | <1 | 2 | >16 | >8 | >4 | >4/76 | <32 | |  |
| HuNan7 | <4 | <4 | <4 | <16 | <1 | 2 | >16 | <1 | <1 | <2/38 | <32 | |  |
| HuNan8 | 16 | <4 | <4 | <16 | <1 | 4 | >16 | 8 | >4 | >4/76 | >128 | |  |
| HuNan9 | <1 | >16 | >16 | <16 | >8 | 4 | >16 | 4 | >4 | >4/76 | <32 | |  |
| HuNan10 | <1 | <4 | <4 | <16 | <1 | 8 | >16 | <1 | <1 | >4/76 | >128 | |  |
| HuNan11 | 4 | >16 | >16 | <16 | <1 | 4 | >16 | <1 | <1 | <2/38 | <32 | |  |
| HuNan12 | 8 | >16 | >16 | <16 | <1 | 4 | >16 | 8 | >4 | >4/76 | 64 | |  |
| HuNan13 | 16 | >16 | >16 | <16 | <1 | <1 | >16 | 8 | >4 | >4/76 | >128 | |  |
| HuNan14 | <1 | <4 | <4 | <16 | >8 | 8 | >16 | <1 | <1 | <2/38 | <32 | |  |
| HuNan15 | 8 | <4 | <4 | <16 | <1 | <1 | >16 | 8 | >4 | >4/76 | <32 | |  |
| HuNan16 | 4 | >16 | >16 | <16 | <1 | 4 | >16 | 8 | >4 | >4/76 | <32 | |  |
| HuNan17 | 4 | >16 | >16 | <16 | <1 | 4 | 8 | <1 | <1 | <2/38 | <32 | |  |
| HuNan18 | <1 | <4 | <4 | <16 | <1 | 8 | >16 | <1 | <1 | >4/76 | <32 | |  |
| HuNan19 | <1 | <4 | <4 | <16 | <1 | 4 | >16 | <1 | <1 | <2/38 | <32 | |  |
| HuNan20 | <1 | <4 | <4 | <16 | <1 | 4 | >16 | 4 | >4 | >4/76 | <32 | |  |
| HuNan21 | >16 | >16 | >16 | <16 | <1 | 2 | >16 | <1 | <1 | >4/76 | <32 | |  |
| HuNan22 | <1 | <4 | <4 | <16 | <1 | 2 | <4 | <1 | <1 | <2/38 | <32 | |  |
| HeNan1 | >16 | <4 | <4 | <16 | <1 | 2 | <4 | >8 | >4 | <2/38 | <32 | |  |
| HeNan2 | <4 | <4 | <4 | <16 | 4 | 8 | >16 | <1 | <1 | >4/76 | <32 | |  |
| HeNan3 | >16 | <4 | <4 | <16 | <1 | 2 | <4 | 8 | >4 | <2/38 | <32 | |  |
| HeNan4 | >16 | <4 | <4 | <16 | <1 | 2 | <4 | >8 | >4 | <2/38 | <32 | |  |
| HeNan5 | >16 | <4 | <4 | <16 | 8 | >16 | >16 | >8 | >4 | <2/38 | <32 | |  |
| HeNan6 | >16 | >16 | >16 | <16 | <1 | >16 | >16 | >8 | >4 | <2/38 | <32 | |  |
| HeNan7 | >16 | <4 | <4 | <16 | <1 | >16 | >16 | 4 | 2 | <2/38 | <32 | |  |
| HeNan8 | 16 | <4 | <4 | <16 | <1 | >16 | >16 | >8 | >4 | <2/38 | 64 | |  |
| HeNan9 | 8 | <4 | <4 | <16 | <1 | 8 | >16 | >8 | >4 | >4/76 | >128 | |  |
| HeNan10 | 8 | <4 | <4 | <16 | <1 | >16 | >16 | >8 | >4 | <2/38 | 64 | |  |
| HeBei1 | >16 | >16 | >16 | <16 | <1 | 4 | >16 | >8 | >4 | >4/76 | <32 | |  |
| HeBei2 | >16 | >16 | >16 | <16 | 4 | 4 | >16 | >8 | >4 | >4/76 | <32 | |  |
| HeBei3 | >16 | >16 | 8 | <16 | >8 | 4 | >16 | >8 | >4 | >4/76 | <32 | |  |
| HeBei4 | >16 | >16 | 8 | <16 | <1 | 4 | >16 | >8 | >4 | >4/76 | <32 | |  |
| HeBei5 | 8 | >16 | >16 | <16 | <1 | 2 | >16 | >8 | >4 | >4/76 | <32 | |  |
| HeBei6 | 2 | >16 | >16 | <16 | <1 | 2 | >16 | >8 | >4 | >4/76 | <32 | |  |
| HeBei7 | 2 | >16 | >16 | <16 | <2 | 2 | >16 | 8 | >4 | >4/76 | <32 | |  |
| HeBei8 | >16 | >16 | >16 | <16 | <1 | 4 | >16 | >8 | >4 | >4/76 | <32 | |  |
| HeBei9 | <1 | <4 | <4 | <16 | <1 | 2 | >16 | <1 | <1 | <2/38 | <32 | |  |
| HeBei10 | 8 | >16 | >16 | <16 | <1 | 4 | >16 | >8 | >4 | >4/76 | <32 | |  |
| HeBei11 | >16 | >16 | >16 | <16 | <2 | >16 | >16 | >8 | >4 | >4/76 | 64 | |  |
| HeBei12 | 16 | >16 | >16 | <16 | 4 | >16 | >16 | 4 | >4 | >4/76 | <32 | |  |
| HeBei13 | >16 | <4 | <4 | <16 | <1 | >16 | >16 | >8 | >4 | >4/76 | <32 | |  |
| HeBei14 | >16 | <4 | <4 | <16 | <1 | 8 | >16 | >8 | >4 | >4/76 | <32 | |  |
| HeBei15 | >16 | <4 | <4 | <16 | <1 | 8 | >16 | >8 | >4 | >4/76 | <32 | |  |

| Strain | Phenotypic AMR | | | | | | | | | | | | |
| --- | --- | --- | --- | --- | --- | --- | --- | --- | --- | --- | --- | --- | --- |
|  | **Monobactams** | **Aminoglycosides** | | | **Tetracyclines** | | | **Quinoloones** | | **Sulfanilamides** | | **Nitrofuran** | |
|  | **ATM** | **GEN** | **TOB** | **AMK** | **TIG** | **MNO** | **TET** | **OFX** | **CIP** | **SXT** | | **NIT** |  |
| HeBei16 | >16 | <4 | <4 | <16 | <1 | <1 | >16 | <1 | <1 | <2/38 | >128 | |  |
| HeBei17 | >16 | <4 | <4 | <16 | <1 | 4 | >16 | >8 | >4 | >4/76 | <32 | |  |
| HeBei18 | 16 | <4 | <4 | <16 | <1 | >16 | >16 | >8 | >4 | >4/76 | <32 | |  |
| HeBei19 | >16 | >16 | >16 | >64 | 4 | >16 | >16 | >8 | >4 | >4/76 | 64 | |  |
| HeBei20 | >16 | >16 | >16 | >64 | <1 | >16 | >16 | >8 | >4 | >4/76 | <32 | |  |
| HeBei21 | >16 | >16 | >16 | <16 | <1 | 4 | >16 | 4 | >4 | >4/76 | <32 | |  |
| HeBei22 | >16 | >16 | >16 | <16 | 4 | 4 | >16 | >8 | >4 | >4/76 | <32 | |  |
| JiangSu1 | >16 | >16 | 8 | <16 | 4 | 4 | >16 | 2 | 2 | >4/76 | <32 | |  |
| JiangSu2 | >16 | >16 | 8 | <16 | 4 | >16 | >16 | >8 | >4 | >4/76 | <32 | |  |
| JiangSu3 | <1 | >16 | >16 | <16 | 4 | >16 | >16 | >8 | >4 | >4/76 | <32 | |  |
| JiangSu4 | <4 | >16 | >16 | <16 | <1 | >16 | >16 | >8 | >4 | >4/76 | <32 | |  |
| JiangSu5 | >16 | >16 | 8 | <16 | 4 | >16 | >16 | >8 | >4 | >4/76 | <32 | |  |
| JiangSu6 | >16 | >16 | 8 | <16 | <1 | >16 | >16 | >8 | >4 | >4/76 | <32 | |  |
| JiangSu7 | >16 | >16 | >16 | <16 | <2 | >16 | >16 | >8 | >4 | >4/76 | <32 | |  |
| JiangSu8 | >16 | >16 | 8 | <16 | <1 | >16 | >16 | 2 | <1 | >4/76 | <32 | |  |
| JiangSu9 | >16 | >16 | 8 | <16 | <1 | 8 | >16 | >8 | >4 | >4/76 | >128 | |  |
| JiangSu10 | >16 | >16 | 8 | <16 | <1 | >16 | >16 | >8 | >4 | >4/76 | <32 | |  |
| JiangSu11 | >16 | >16 | >16 | >64 | 4 | 8 | >16 | >8 | >4 | >4/76 | <32 | |  |
| JiangSu12 | >16 | >16 | >16 | >64 | <2 | >16 | >16 | >8 | >4 | >4/76 | <32 | |  |
| JiangSu13 | >16 | >16 | >16 | <16 | <1 | 4 | >16 | 8 | >4 | >4/76 | <32 | |  |
| JiangSu14 | >16 | >16 | 8 | <16 | >8 | 8 | >16 | >8 | >4 | >4/76 | <32 | |  |
| JiangSu15 | 16 | >16 | >16 | <16 | <1 | <1 | >16 | >8 | >4 | >4/76 | <32 | |  |
| JiangSu16 | >16 | >16 | >16 | >64 | <1 | >16 | >16 | >8 | >4 | >4/76 | <32 | |  |
| JiangSu17 | 16 | >16 | >16 | <16 | >8 | 8 | >16 | >8 | >4 | >4/76 | <32 | |  |
| JiangSu18 | >16 | >16 | >16 | >64 | <1 | >16 | >16 | >8 | >4 | >4/76 | <32 | |  |
| FuJian2 | <4 | <4 | <4 | <16 | <1 | >16 | >16 | >8 | >4 | >4/76 | >128 | |  |
| FuJian3 | >16 | <4 | <4 | <16 | 4 | >16 | >16 | 2 | <1 | >4/76 | <32 | |  |
| FuJian4 | <1 | <4 | <4 | <16 | <1 | >16 | >16 | >8 | >4 | >4/76 | 64 | |  |
| FuJian5 | 16 | <4 | <4 | <16 | <1 | 4 | >16 | <1 | <1 | <2/38 | 64 | |  |
| FuJian6 | 16 | <4 | <4 | <16 | <1 | 4 | >16 | <1 | <1 | <2/38 | 64 | |  |
| FuJian7 | >16 | <4 | <4 | <16 | <1 | 4 | >16 | 2 | 2 | >4/76 | <32 | |  |
| NingXia1 | >16 | >16 | >16 | >64 | <1 | >16 | >16 | >8 | >4 | >4/76 | >128 | |  |
| NingXia2 | >16 | >16 | >16 | >64 | <1 | >16 | >16 | >8 | >4 | >4/76 | >128 | |  |
| NingXia3 | >16 | >16 | >16 | >64 | <1 | >16 | >16 | >8 | >4 | >4/76 | 64 | |  |
| NingXia4 | >16 | >16 | >16 | >64 | <1 | >16 | >16 | >8 | >4 | >4/76 | >128 | |  |
| NingXia5 | >16 | >16 | >16 | >64 | <1 | 8 | >16 | >8 | >4 | >4/76 | >128 | |  |
| NingXia6 | 16 | >16 | >16 | >64 | <1 | 8 | >16 | >8 | >4 | >4/76 | 64 | |  |
| NingXia7 | 16 | >16 | >16 | >64 | <1 | 8 | >16 | >8 | 2 | >4/76 | 64 | |  |
| NingXia8 | >16 | >16 | >16 | >64 | <1 | 8 | >16 | 8 | >4 | <2/38 | <32 | |  |
| NingXia9 | >16 | >16 | >16 | >64 | <1 | >16 | >16 | >8 | >4 | >4/76 | >128 | |  |
| NingXia10 | >16 | >16 | >16 | >64 | <1 | 8 | >16 | >8 | >4 | >4/76 | 64 | |  |
| NingXia11 | >16 | >16 | >16 | >64 | <1 | 8 | >16 | >8 | >4 | >4/76 | >128 | |  |
| ShaanXi1 | >16 | <4 | <4 | <16 | 4 | >16 | >16 | <1 | <1 | >4/76 | <32 | |  |
| ShaanXi2 | <4 | >16 | >16 | <16 | 4 | 8 | >16 | >8 | >4 | >4/76 | 64 | |  |
| ShaanXi3 | >16 | >16 | 8 | <16 | <1 | >16 | >16 | <1 | <1 | >4/76 | <32 | |  |
| ShaanXi4 | 16 | <4 | <4 | <16 | <1 | >16 | >16 | >8 | >4 | >4/76 | <32 | |  |
| ShaanXi5 | 16 | >16 | >16 | >64 | <1 | >16 | >16 | >8 | >4 | >4/76 | <32 | |  |
| ShaanXi6 | >16 | >16 | >16 | >64 | <1 | >16 | >16 | >8 | >4 | >4/76 | <32 | |  |

| Strain | Plasmid replicons | | | | | | | | | | | | | | | | | | |
| --- | --- | --- | --- | --- | --- | --- | --- | --- | --- | --- | --- | --- | --- | --- | --- | --- | --- | --- | --- |
|  | **Number** | | **28** | **49** | **4** | **12** | **3** | **68** | **1** | **1** | **2** | **28** | **5** | **42** | **42** | **26** | **17** | **13** | |
|  | **Types** | | **IncFII (pHN7A8)HN7A8)** | **IncI1-I(Alpha)** | **IncY** | **IncFIA** | **IncFIA**  **(HI1)** | **IncFIB**  **(AP001918)** | **Inc**  **HI1A** | **IncHI1B**  **(R27)** | **IncI2**  **(Delta)** | **p0111** | **IncFII (pRSB107)** | **IncHI2** | **IncHI2A** | **IncX1** | **IncFII**  **(pCoo)** | **IncI2** |  |
| HuNan1 |  | |  | * |  |  |  | * |  |  |  |  |  | * | * |  |  |  |  |
| HuNan2 |  | |  | * |  |  |  | * |  |  |  |  |  | * | * |  |  |  |  |
| HuNan3 |  | |  |  |  | * |  | * |  |  |  | * |  | * | * |  |  |  |  |
| HuNan4 |  | | * |  |  | * |  | * |  |  |  | * |  |  |  |  |  |  |  |
| HuNan5 |  | |  | * |  |  |  |  |  |  |  | * |  |  |  |  |  |  |  |
| HuNan6 |  | |  | * |  |  |  | * |  |  |  | * |  |  |  |  |  | * |  |
| HuNan7 |  | |  |  |  |  |  | * |  |  |  | * |  |  |  |  |  |  |  |
| HuNan8 |  | |  | * |  |  |  | * |  |  |  | * |  |  |  |  |  | * |  |
| HuNan9 |  | |  |  |  |  |  |  |  |  |  |  |  |  |  | * |  |  |  |
| HuNan10 | |  |  | * |  |  |  |  |  |  |  |  |  |  |  |  |  |  |  |
| HuNan11 | |  |  | * |  |  |  | * |  |  |  |  |  | * | * |  |  |  |  |
| HuNan12 | |  |  |  |  |  |  |  |  |  |  | * |  |  |  |  |  |  |  |
| HuNan13 | |  |  |  |  | * |  | * |  |  |  | * |  | * | * |  |  |  |  |
| HuNan14 | |  |  |  |  |  |  |  |  |  |  |  |  |  |  |  |  |  |  |
| HuNan15 | |  |  | * |  |  |  | * |  |  |  | * |  |  |  |  |  | * |  |
| HuNan16 | |  |  |  |  |  |  | * |  |  |  |  |  |  |  |  |  |  |  |
| HuNan17 | |  |  | * |  |  |  | * |  |  |  |  |  | * | * |  |  |  |  |
| HuNan18 | |  |  | * |  |  |  |  |  |  |  |  |  |  |  |  |  |  |  |
| HuNan19 | |  |  | * |  |  |  | * |  |  |  |  |  |  |  | * | * |  |  |
| HuNan20 | |  |  |  | * | * |  | * |  |  |  |  |  | * | * |  |  |  |  |
| HuNan21 | |  |  |  |  |  |  | * |  |  |  | * |  |  |  | * |  |  |  |
| HuNan22 | |  |  |  | * |  |  |  |  |  |  |  |  |  |  |  |  |  |  |
| HeNan1 | |  |  |  |  |  |  |  |  |  |  |  |  |  |  |  |  | * |  |
| HeNan2 | |  |  | * |  |  |  | * |  |  |  |  |  |  |  |  |  |  |  |
| HeNan3 | |  |  | * |  |  |  |  |  |  |  |  |  |  |  |  | * | * |  |
| HeNan4 | |  |  | * |  |  |  |  |  |  |  |  |  |  |  |  |  | * |  |
| HeNan5 | |  | * | * |  | * | * | * | * | * | * | * |  |  |  |  |  |  |  |
| HeNan6 | |  | * | * | * |  |  |  |  |  |  |  |  |  |  |  |  |  |  |
| HeNan7 | |  | * |  |  | * |  | * |  |  |  |  |  |  |  | * |  |  |  |
| HeNan8 | |  | * |  |  |  |  | * |  |  |  |  |  |  |  | * |  |  |  |
| HeNan9 | |  |  |  |  |  |  | * |  |  |  | * |  | * | * | * |  |  |  |
| HeNan10 | |  | * |  |  |  |  | * |  |  |  |  |  |  |  | * |  |  |  |
| HeBei1 | |  | * | * |  |  |  | * |  |  |  |  |  |  |  |  |  |  |  |
| HeBei2 | |  | * | * |  |  |  | * |  |  |  |  |  |  |  |  |  |  |  |
| HeBei3 | |  | * | * |  |  |  | * |  |  |  |  |  |  |  |  |  |  |  |
| HeBei4 | |  | * | * |  |  |  | * |  |  |  |  |  |  |  |  |  |  |  |
| HeBei5 | |  |  | * |  |  |  |  |  |  |  |  |  | * | * |  |  | * |  |
| HeBei6 | |  |  | * |  |  |  |  |  |  | * |  |  |  |  |  |  |  |  |
| HeBei7 | |  |  | * |  |  |  |  |  |  |  |  |  |  |  |  |  |  |  |
| HeBei8 | |  |  | * |  |  |  |  |  |  |  | * |  |  |  |  |  |  |  |
| HeBei9 | |  |  | * |  |  |  | * |  |  |  |  |  |  |  |  |  |  |  |
| HeBei10 | |  |  | * |  |  |  |  |  |  |  |  |  | * | * |  |  |  |  |
| HeBei11 | |  |  | * |  |  |  |  |  |  |  |  |  | * | * | * | * |  |  |
| HeBei12 | |  |  | * |  |  |  |  |  |  |  |  |  | * | * | * |  |  |  |
| HeBei13 | |  |  | * |  |  |  |  |  |  |  | * |  |  |  | * |  |  |  |
| HeBei14 | |  | * | * |  |  |  |  |  |  |  | * |  |  |  |  |  |  |  |
| HeBei15 | |  | * | * |  |  |  |  |  |  |  | * |  |  |  |  |  |  |  |

| Strain | Plasmid replicons | | | | | | | | | | | | | | | | | | | |
| --- | --- | --- | --- | --- | --- | --- | --- | --- | --- | --- | --- | --- | --- | --- | --- | --- | --- | --- | --- | --- |
|  | **Number** | | | **28** | **49** | **4** | **12** | **3** | **68** | **1** | **1** | **2** | **28** | **5** | **42** | **42** | **26** | **17** | **13** | |
|  | **Types** | | | **IncFII (pHN7A8)HN7A8)** | **IncI1-I(Alpha)** | **IncY** | **IncFIA** | **IncFIA**  **(HI1)** | **IncFIB**  **(AP001918)** | **Inc**  **HI1A** | **IncHI1B**  **(R27)** | **IncI2**  **(Delta)** | **p0111** | **IncFII (pRSB107)** | **IncHI2** | **IncHI2A** | **IncX1** | **IncFII**  **(pCoo)** | **IncI2** |  |
| HeBei16 | |  | |  |  |  |  |  | * |  |  |  | * |  |  |  | * |  |  |  |
| HeBei17 | |  | |  | * |  |  |  | * |  |  |  |  |  |  |  | * |  |  |  |
| HeBei18 | |  | | * | * |  |  |  | * |  |  |  | * |  | * | * |  |  |  |  |
| HeBei19 | |  | |  |  |  | * |  | * |  |  |  |  |  | * | * | * | * |  |  |
| HeBei20 | |  | | * | * |  |  |  | * |  |  |  |  |  | * | * |  | * | * |  |
| HeBei21 | |  | | * |  |  |  |  |  |  |  |  |  |  | * | * |  |  |  |  |
| HeBei22 | |  | |  |  | * |  |  |  |  |  |  |  |  | * | * |  |  |  |  |
| JiangSu1 | | |  |  |  |  |  |  | * |  |  |  |  |  | * | * |  | * |  |  |
| JiangSu2 | | |  |  |  |  |  |  | * |  |  |  |  |  | * | * |  | * |  |  |
| JiangSu3 | | |  |  |  |  |  |  | * |  |  |  |  |  | * | * |  |  |  |  |
| JiangSu4 | | |  |  | * |  |  |  | * |  |  |  | * |  | * | * |  |  |  |  |
| JiangSu5 | | |  |  | * |  |  |  | * |  |  |  |  | * | * | * |  | * |  |  |
| JiangSu6 | | |  |  | * |  |  |  | * |  |  |  |  | * | * | * |  |  |  |  |
| JiangSu7 | | |  |  | * |  |  |  | * |  |  |  |  |  | * | * |  | * |  |  |
| JiangSu8 | | |  |  | * |  |  |  | * |  |  |  |  |  | * | * |  | * |  |  |
| JiangSu9 | | |  |  |  |  |  |  | * |  |  |  | * |  | * | * |  |  |  |  |
| JiangSu10 | | |  |  | * |  |  |  | * |  |  |  |  | * | * | * |  | * |  |  |
| JiangSu11 | | |  |  |  |  |  |  | * |  |  |  | * |  |  |  | * |  |  |  |
| JiangSu12 | | |  |  |  |  |  |  | * |  |  |  | * |  |  |  | * |  |  |  |
| JiangSu13 | | |  | * | * |  |  |  | * |  |  |  |  |  |  |  | * |  |  |  |
| JiangSu14 | | |  |  | * |  |  |  | * |  |  |  |  |  | * | * |  |  |  |  |
| JiangSu15 | | |  |  | * |  |  |  |  |  |  |  | * |  | * | * |  |  |  |  |
| JiangSu16 | | |  |  |  |  |  |  | * |  |  |  |  |  |  |  | * |  |  |  |
| JiangSu17 | | |  |  | * |  |  |  | * |  |  |  |  |  | * | * |  |  |  |  |
| JiangSu18 | | |  |  |  |  |  |  | * |  |  |  |  |  |  |  | * |  |  |  |
| FuJian2 | | |  |  |  |  |  | * | * |  |  |  |  |  | * | * |  |  |  |  |
| FuJian3 | | |  |  |  |  | * |  | * |  |  |  |  |  | * | * | * | * |  |  |
| FuJian4 | | |  |  |  |  |  | * | * |  |  |  |  |  | * | * |  |  |  |  |
| FuJian5 | | |  | * |  |  |  |  | * |  |  |  |  |  |  |  | * |  |  |  |
| FuJian6 | | |  | * |  |  |  |  | * |  |  |  |  |  |  |  | * |  |  |  |
| FuJian7 | | |  | * | * |  |  |  | * |  |  |  |  |  | * | * |  |  |  |  |
| NingXia1 | | |  |  | * |  | * |  | * |  |  |  |  |  | * | * |  |  |  |  |
| NingXia2 | | |  |  | * |  | * |  | * |  |  |  |  |  | * | * |  |  |  |  |
| NingXia3 | | |  |  | * |  | * |  | * |  |  |  |  |  | * | * |  |  |  |  |
| NingXia4 | | |  |  | * |  | * |  | * |  |  |  |  |  | * | * |  |  |  |  |
| NingXia5 | | |  | * |  |  |  |  | * |  |  |  | * |  |  |  |  | * | * |  |
| NingXia6 | | |  | * |  |  |  |  | * |  |  |  | * |  |  |  |  | * | * |  |
| NingXia7 | | |  | * |  |  |  |  | * |  |  |  | * |  |  |  |  | * | * |  |
| NingXia8 | | |  | * |  |  |  |  | * |  |  |  |  |  | * | * |  | * | * |  |
| NingXia9 | | |  | * |  |  |  |  | * |  |  |  | * |  |  |  |  | * | * |  |
| NingXia10 | | |  | * |  |  |  |  | * |  |  |  |  |  |  |  | * |  |  |  |
| NingXia11 | | |  | * |  |  |  |  | * |  |  |  |  |  |  |  | * |  |  |  |
| ShaanXi1 | | |  |  |  |  |  |  |  |  |  |  | * |  | * | * | * |  |  |  |
| ShaanXi2 | | |  |  |  |  |  |  | * |  |  |  |  | * | * | * |  |  |  |  |
| ShaanXi3 | | |  |  |  |  |  |  | * |  |  |  |  |  | * | * |  |  |  |  |
| ShaanXi4 | | |  |  |  |  |  |  | * |  |  |  |  | * |  |  |  |  |  |  |
| ShaanXi5 | | |  | * |  |  |  |  |  |  |  |  |  |  |  |  | * |  |  |  |
| ShaanXi6 | | |  | * |  |  |  |  |  |  |  |  |  |  |  |  | * |  |  |  |

| Strain | Plasmid replicons | | | | | | | | | | | | | | | | |  | |
| --- | --- | --- | --- | --- | --- | --- | --- | --- | --- | --- | --- | --- | --- | --- | --- | --- | --- | --- | --- |
|  | **Number** | **12** | **2** | **1** | **6** | **3** | **7** | **14** | **5** | **6** | **9** | **2** | **3** | **1** | **1** | **1** | **1** | **5** | |
|  | **Types** | **IncN** | **IncFIB(H89-PhagePlasmid)** | **IncQ1** | **IncFII**  **(29)** | **ColpVC** | **Col156** | **IncFIC(FII)** | **IncFII** | **IncFII**  **(pSFO)** | **IncR** | **IncX4** | **IncFIB**  **(K)** | **Col(pHAD28)** | **Col440I** | **IncFIB(pLF82-PhagePlasmid)** | **IncI3(Delta)** | **Col(MG828)** |  |
| HuNan1 |  |  |  |  |  |  |  |  |  |  |  |  |  |  |  |  |  |  |  |
| HuNan2 |  |  |  |  |  |  |  |  | * |  |  |  |  |  |  |  |  |  |  |
| HuNan3 |  |  |  |  |  |  |  | * |  |  |  |  |  |  |  |  |  | * |  |
| HuNan4 |  |  |  |  |  |  |  |  | * |  |  |  |  |  |  |  |  |  |  |
| HuNan5 |  |  |  |  |  |  | * |  |  |  |  |  |  |  |  |  |  |  |  |
| HuNan6 |  |  |  |  |  |  | * | * |  |  |  |  |  |  |  |  |  |  |  |
| HuNan7 |  |  |  |  |  |  |  |  |  |  |  |  |  |  |  |  |  |  |  |
| HuNan8 |  |  |  |  |  |  | * | * |  |  |  |  |  |  |  |  |  |  |  |
| HuNan9 |  |  |  |  |  |  |  |  |  |  |  |  |  |  |  |  |  |  |  |
| HuNan10 |  |  |  |  |  |  |  |  |  |  |  |  |  |  |  |  |  |  |  |
| HuNan11 |  |  |  |  |  |  |  |  |  |  |  |  |  |  |  |  |  |  |  |
| HuNan12 |  |  |  |  |  |  |  |  |  |  |  |  | * |  |  |  |  |  |  |
| HuNan13 |  |  |  |  |  |  |  | * |  |  |  |  |  |  |  |  |  | * |  |
| HuNan14 |  |  |  |  |  |  |  |  |  |  |  |  |  |  |  |  |  |  |  |
| HuNan15 |  |  |  |  |  |  | * | * |  |  |  |  |  |  |  |  |  |  |  |
| HuNan16 |  |  |  |  |  |  |  |  | * |  |  |  |  |  |  |  |  |  |  |
| HuNan17 |  |  |  |  |  |  |  |  |  |  |  |  |  |  |  |  |  |  |  |
| HuNan18 |  |  |  |  |  |  |  |  |  |  |  |  |  |  |  |  |  |  |  |
| HuNan19 |  |  |  |  |  |  |  |  |  |  |  |  |  |  |  |  |  |  |  |
| HuNan20 |  |  |  |  |  |  |  | * |  |  |  |  |  |  |  |  |  | * |  |
| HuNan21 |  |  |  |  |  |  |  |  |  |  |  | * |  |  |  |  |  |  |  |
| HuNan22 |  |  |  |  |  |  |  |  |  |  |  |  |  |  |  |  |  |  |  |
| HeNan1 |  |  |  |  |  |  |  |  |  |  |  |  |  |  |  |  |  |  |  |
| HeNan2 |  |  |  |  |  |  |  |  | * |  |  |  |  |  |  |  |  |  |  |
| HeNan3 |  |  |  |  |  |  | * |  |  |  |  | * |  |  |  |  |  |  |  |
| HeNan4 |  |  |  |  |  |  |  |  |  |  |  |  |  |  |  |  |  |  |  |
| HeNan5 |  |  |  |  |  |  |  |  |  |  |  |  |  |  |  |  |  |  |  |
| HeNan6 |  |  |  |  |  |  |  |  |  |  |  |  |  |  |  |  |  |  |  |
| HeNan7 |  |  |  |  | * |  |  |  |  |  |  |  |  |  |  |  |  |  |  |
| HeNan8 |  |  |  |  |  |  |  |  |  |  |  |  |  |  |  |  |  |  |  |
| HeNan9 |  |  |  |  |  | * |  |  |  |  |  |  |  |  |  |  |  |  |  |
| HeNan10 |  |  |  |  |  |  |  |  |  |  |  |  |  |  |  |  |  |  |  |
| HeBei1 |  |  |  |  |  |  |  |  |  |  |  |  |  |  |  |  |  |  |  |
| HeBei2 |  |  |  |  |  |  |  |  |  |  |  |  |  |  |  |  |  |  |  |
| HeBei3 |  |  |  |  |  |  |  |  |  |  |  |  |  |  |  |  |  |  |  |
| HeBei4 |  |  |  |  |  |  |  |  |  |  |  |  |  |  |  |  |  |  |  |
| HeBei5 |  |  |  |  |  |  |  |  |  |  |  |  | * |  |  |  |  |  |  |
| HeBei6 |  |  |  |  |  |  |  |  |  |  |  |  |  |  |  |  |  |  |  |
| HeBei7 |  |  |  |  |  |  |  |  |  |  |  |  |  |  |  |  | * |  |  |
| HeBei8 |  |  |  |  |  |  |  |  |  |  |  |  |  |  |  |  |  |  |  |
| HeBei9 |  |  |  |  |  |  |  |  |  |  |  |  |  |  |  |  |  |  |  |
| HeBei10 |  |  |  |  |  |  |  |  |  |  | * |  |  |  |  |  |  |  |  |
| HeBei11 |  |  |  |  |  |  |  |  |  |  |  |  |  |  |  | * |  |  |  |
| HeBei12 |  |  |  |  |  |  |  |  |  |  |  |  |  |  |  |  |  |  |  |
| HeBei13 |  |  |  |  |  |  |  |  |  |  |  |  |  |  |  |  |  |  |  |
| HeBei14 |  |  |  |  |  | * |  |  |  |  |  |  |  |  |  |  |  |  |  |
| HeBei15 |  |  |  |  |  | * |  |  |  |  |  |  |  | * | * |  |  |  |  |

| Strain | Plasmid replicons | | | | | | | | | | | | | | | | | |  | |
| --- | --- | --- | --- | --- | --- | --- | --- | --- | --- | --- | --- | --- | --- | --- | --- | --- | --- | --- | --- | --- |
|  | **Number** | | **12** | **2** | **1** | **6** | **3** | **7** | **14** | **5** | **6** | **9** | **2** | **3** | **1** | **1** | **1** | **1** | **5** | |
|  | **Types** | | **IncN** | **IncFIB(H89-PhagePlasmid)** | **IncQ1** | **IncFII**  **(29)** | **ColpVC** | **Col156** | **IncFIC(FII)** | **IncFII** | **IncFII**  **(pSFO)** | **IncR** | **IncX4** | **IncFIB**  **(K)** | **Col(pHAD28)** | **Col440I** | **IncFIB(pLF82-PhagePlasmid)** | **IncI3(Delta)** | **Col(MG828)** |  |
| HeBei16 |  | |  |  |  |  |  | * |  |  |  |  |  |  |  |  |  |  |  |  |
| HeBei17 |  | | * |  |  |  |  |  |  | * |  |  |  |  |  |  |  |  |  |  |
| HeBei18 |  | |  |  |  |  |  |  | * |  |  |  |  |  |  |  |  |  |  |  |
| HeBei19 |  | |  | * |  |  |  |  |  |  |  |  |  |  |  |  |  |  |  |  |
| HeBei20 |  | |  |  |  |  |  |  |  |  |  |  |  |  |  |  |  |  |  |  |
| HeBei21 |  | |  |  |  |  |  |  |  |  |  |  |  |  |  |  |  |  |  |  |
| HeBei22 |  | |  |  |  |  |  |  |  |  |  |  |  |  |  |  |  |  |  |  |
| JiangSu1 |  | |  |  |  |  |  |  |  |  |  |  |  |  |  |  |  |  |  |  |
| JiangSu2 |  | |  |  |  |  |  |  |  |  |  |  |  |  |  |  |  |  |  |  |
| JiangSu3 |  | |  |  |  |  |  |  |  |  |  | * |  |  |  |  |  |  |  |  |
| JiangSu4 |  | |  |  |  |  |  |  |  |  |  | * |  |  |  |  |  |  |  |  |
| JiangSu5 |  | |  |  |  |  |  |  |  |  |  |  |  |  |  |  |  |  |  |  |
| JiangSu6 |  | |  |  |  |  |  |  |  |  |  |  |  |  |  |  |  |  |  |  |
| JiangSu7 |  | |  |  |  |  |  |  |  |  | * |  |  |  |  |  |  |  |  |  |
| JiangSu8 |  | |  |  |  |  |  |  |  |  |  |  |  |  |  |  |  |  |  |  |
| JiangSu9 |  | |  |  |  |  |  |  |  |  | * |  |  |  |  |  |  |  |  |  |
| JiangSu10 | |  |  |  |  |  |  |  |  |  |  |  |  |  |  |  |  |  |  |  |
| JiangSu11 | |  |  |  |  |  |  |  | * |  |  |  |  |  |  |  |  |  |  |  |
| JiangSu12 | |  |  |  |  |  |  |  | * |  |  |  |  |  |  |  |  |  |  |  |
| JiangSu13 | |  | * |  |  |  |  | * | * |  |  |  |  |  |  |  |  |  |  |  |
| JiangSu14 | |  |  |  |  |  |  |  | * |  |  |  |  |  |  |  |  |  |  |  |
| JiangSu15 | |  | * |  |  |  |  |  |  |  |  |  |  | * |  |  |  |  |  |  |
| JiangSu16 | |  |  |  |  |  |  |  | * |  |  |  |  |  |  |  |  |  |  |  |
| JiangSu17 | |  |  |  |  |  |  |  | * |  |  |  |  |  |  |  |  |  |  |  |
| JiangSu18 | |  |  |  |  |  |  |  | * |  |  |  |  |  |  |  |  |  |  |  |
| FuJian2 | |  |  |  |  |  |  |  |  |  | * | * |  |  |  |  |  |  | * |  |
| FuJian3 | |  |  | * | * |  |  |  |  |  |  |  |  |  |  |  |  |  |  |  |
| FuJian4 | |  |  |  |  |  |  |  |  |  | * | * |  |  |  |  |  |  | * |  |
| FuJian5 | |  | * |  |  |  |  |  |  |  |  |  |  |  |  |  |  |  |  |  |
| FuJian6 | |  | * |  |  |  |  |  |  |  |  |  |  |  |  |  |  |  |  |  |
| FuJian7 | |  |  |  |  |  |  |  |  |  |  |  |  |  |  |  |  |  |  |  |
| NingXia1 | |  |  |  |  | * |  |  |  |  |  | * |  |  |  |  |  |  |  |  |
| NingXia2 | |  |  |  |  | * |  |  |  |  |  | * |  |  |  |  |  |  |  |  |
| NingXia3 | |  |  |  |  | * |  |  |  |  |  | * |  |  |  |  |  |  |  |  |
| NingXia4 | |  |  |  |  | * |  |  |  |  |  | * |  |  |  |  |  |  |  |  |
| NingXia5 | |  | * |  |  |  |  |  |  |  |  |  |  |  |  |  |  |  |  |  |
| NingXia6 | |  | * |  |  |  |  |  |  |  |  |  |  |  |  |  |  |  |  |  |
| NingXia7 | |  | * |  |  |  |  |  |  |  |  |  |  |  |  |  |  |  |  |  |
| NingXia8 | |  | * |  |  |  |  |  |  |  |  |  |  |  |  |  |  |  |  |  |
| NingXia9 | |  | * |  |  |  |  |  |  |  |  |  |  |  |  |  |  |  |  |  |
| NingXia10 | |  |  |  |  |  |  |  |  |  | * |  |  |  |  |  |  |  |  |  |
| NingXia11 | |  |  |  |  |  |  |  |  |  | * |  |  |  |  |  |  |  |  |  |
| ShaanXi1 | |  |  |  |  | * |  |  |  |  |  |  |  |  |  |  |  |  |  |  |
| ShaanXi2 | |  |  |  |  |  |  |  |  |  |  |  |  |  |  |  |  |  |  |  |
| ShaanXi3 | |  | * |  |  |  |  |  |  |  |  |  |  |  |  |  |  |  |  |  |
| ShaanXi4 | |  |  |  |  |  |  |  |  |  |  |  |  |  |  |  |  |  |  |  |
| ShaanXi5 | |  | * |  |  |  |  |  |  |  |  |  |  |  |  |  |  |  |  |  |
| ShaanXi6 | |  |  |  |  |  |  |  |  |  |  |  |  |  |  |  |  |  |  |  |

| Strain | ARGs | Beta-lactam | | | | | | | | | | | | | | | Sulfonamide | | | | | | | | | | |
| --- | --- | --- | --- | --- | --- | --- | --- | --- | --- | --- | --- | --- | --- | --- | --- | --- | --- | --- | --- | --- | --- | --- | --- | --- | --- | --- | --- |
|  | **Number** | | **95** | **9** | | **52** | **2** | **20** | | **1** | **60** | **4** | **3** | **24** | **12** | **2** | | **16** | **62** | **32** | **3** | **2** | **6** | **16** | **46** | **10** | |
|  | **Types** | | ***bla***  **EC** | ***bla*CTX-M-14** | | ***bla*CTX-M-55** | ***bla*CTX-M-64** | ***bla*CTX-M-65** | | ***bla*CTX-M-121** | ***bla*TEM-1** | ***bla*TEM-135** | ***bla*OXA-1** | ***bla*OXA-10** | ***bla*LAP-2** | ***bla*CMY-2** | | ***sul*1** | ***sul*2** | ***sul*3** | ***dfr*A1** | ***dfr*A5** | ***dfr*A8** | ***dfr*A12** | ***dfr*A14** | ***dfr*A17** |  |
| HuNan1 |  | | * | | * |  |  | |  |  |  |  |  |  |  |  | |  | * | * |  |  |  |  |  |  |  |
| HuNan2 |  | | * | |  |  |  | | * |  |  |  |  | * |  |  | |  |  | * |  |  |  |  | * |  |  |
| HuNan3 |  | | * | |  |  |  | | * |  |  |  |  |  |  |  | |  | * |  |  |  |  |  | * |  |  |
| HuNan4 |  | | * | |  | * |  | |  |  | * |  |  |  |  |  | |  |  |  |  |  |  |  |  |  |  |
| HuNan5 |  | | * | |  |  |  | | * |  |  |  |  |  |  |  | | * | * |  |  |  |  |  |  | * |  |
| HuNan6 |  | | * | |  |  |  | | * |  |  |  |  |  |  |  | | * | * |  |  |  |  |  |  | * |  |
| HuNan7 |  | | * | |  |  |  | |  |  | * |  |  |  |  |  | |  |  |  | * |  |  |  |  |  |  |
| HuNan8 |  | | * | |  |  |  | | * |  |  |  |  |  |  |  | | * | * |  |  |  |  |  |  | * |  |
| HuNan9 |  | | * | |  |  |  | |  |  | * |  |  |  |  |  | |  | * |  |  |  |  |  | * |  |  |
| HuNan10 |  | | * | |  |  |  | |  |  |  |  |  | * |  |  | |  |  |  |  |  |  |  | * |  |  |
| HuNan11 |  | | * | | * |  |  | |  |  |  |  |  |  |  |  | |  | * | * |  |  |  |  |  |  |  |
| HuNan12 |  | | * | |  |  |  | | * |  |  |  |  | * |  |  | |  | * |  |  |  |  | * |  |  |  |
| HuNan13 |  | | * | |  |  |  | | * |  | * |  |  |  |  |  | |  | * |  |  |  |  |  |  |  |  |
| HuNan14 |  | | * | |  |  |  | |  |  |  |  |  |  |  |  | |  |  |  |  |  |  |  |  |  |  |
| HuNan15 |  | | * | |  |  |  | | * |  |  |  |  |  |  |  | | * | * |  |  |  |  |  |  | * |  |
| HuNan16 |  | | * | |  |  |  | |  |  | * |  |  |  |  |  | |  | * |  |  |  |  |  |  | * |  |
| HuNan17 |  | | * | | * |  |  | |  |  |  |  |  |  |  |  | |  | * | * |  |  |  |  |  |  |  |
| HuNan18 |  | | * | |  |  |  | |  |  |  |  |  | * |  |  | |  |  |  |  |  |  |  | * |  |  |
| HuNan19 |  | | * | |  |  |  | |  |  |  |  |  | * |  |  | |  |  |  |  |  |  |  |  |  |  |
| HuNan20 |  | | * | |  |  |  | |  |  | * |  |  |  |  |  | |  | * |  |  |  |  |  | * |  |  |
| HuNan21 |  | | * | |  |  |  | | * |  | * |  |  |  |  |  | | * | * |  |  |  |  |  |  | * |  |
| HuNan22 |  | | * | |  |  |  | |  |  | * |  |  |  |  |  | |  | * |  |  |  |  |  |  |  |  |
| HeNan1 |  | | * | |  |  | * | |  |  |  |  |  |  |  |  | |  |  |  |  |  |  |  |  |  |  |
| HeNan2 |  | | * | |  |  |  | |  |  |  | * |  |  |  |  | |  |  |  |  |  |  |  | * |  |  |
| HeNan3 |  | | * | |  | * |  | |  |  | * |  |  |  |  |  | |  |  |  |  |  |  |  |  |  |  |
| HeNan4 |  | | * | |  |  | * | |  |  |  |  |  |  |  |  | |  |  |  |  |  |  |  |  |  |  |
| HeNan5 |  | | * | |  | * |  | |  | * | * |  |  |  |  |  | |  | * |  |  |  |  |  |  |  |  |
| HeNan6 |  | | * | |  | * |  | | * |  | * |  |  |  |  |  | |  | * |  |  |  |  |  |  |  |  |
| HeNan7 |  | | * | |  |  |  | |  |  |  |  |  |  |  |  | |  |  |  |  |  |  |  |  |  |  |
| HeNan8 |  | | * | |  | * |  | |  |  | * |  |  |  |  |  | |  | * |  |  |  |  |  |  |  |  |
| HeNan9 |  | | * | |  |  |  | | * |  |  |  |  | * |  |  | |  |  |  |  |  |  |  | * |  |  |
| HeNan10 |  | | * | |  | * |  | |  |  | * |  |  |  |  |  | |  | * |  |  |  |  |  |  |  |  |
| HeBei1 |  | | * | |  | * |  | |  |  |  |  |  |  |  |  | |  |  | * |  |  |  |  | * |  |  |
| HeBei2 |  | | * | |  | * |  | |  |  |  |  |  |  |  |  | |  |  | * |  |  |  |  | * |  |  |
| HeBei3 |  | | * | |  | * |  | |  |  |  |  |  |  |  |  | |  |  | * |  |  |  |  | * |  |  |
| HeBei4 |  | | * | |  | * |  | |  |  |  |  |  |  |  |  | |  |  | * |  |  |  |  | * |  |  |
| HeBei5 |  | | * | | * |  |  | |  |  |  |  |  |  |  |  | |  | * |  |  |  |  | * |  | * |  |
| HeBei6 |  | | * | | * |  |  | |  |  |  |  |  |  |  |  | | * | * |  | * |  |  |  |  |  |  |
| HeBei7 |  | | * | | * | * |  | |  |  |  |  |  |  |  |  | | * | * |  | * |  |  |  |  |  |  |
| HeBei8 |  | | * | | * |  |  | |  |  |  |  |  |  |  |  | |  | * |  |  |  |  |  |  | * |  |
| HeBei9 |  | | * | |  |  |  | |  |  |  |  |  | * |  |  | |  |  |  |  |  |  |  | * |  |  |
| HeBei10 |  | | * | |  |  |  | | * |  | * |  |  | * |  |  | |  |  | * |  |  |  | * |  |  |  |
| HeBei11 |  | | * | |  | * |  | | * |  | * |  | * | * |  |  | | * | * |  |  |  |  | * | * |  |  |
| HeBei12 |  | | * | |  |  |  | | * |  |  |  | * |  |  |  | | * | * |  |  |  |  | * |  |  |  |
| HeBei13 |  | | * | |  | * |  | |  |  | * |  |  | * |  |  | |  |  |  |  |  |  |  | * |  |  |
| HeBei14 |  | | * | |  | * |  | |  |  | * |  |  |  |  |  | |  |  | * |  |  |  | * |  |  |  |
| HeBei15 |  | | * | |  | * |  | |  |  | * |  |  |  |  |  | |  | * | * |  |  |  | * |  |  |  |

| Strain | ARGs | | Beta-lactam | | | | | | | | | | | | | | | Sulfonamide | | | | | | | | | | |
| --- | --- | --- | --- | --- | --- | --- | --- | --- | --- | --- | --- | --- | --- | --- | --- | --- | --- | --- | --- | --- | --- | --- | --- | --- | --- | --- | --- | --- |
|  | **Number** | | | **95** | **9** | | **52** | **2** | | **20** | **1** | **60** | **4** | **3** | **24** | **12** | **2** | | **16** | **62** | **32** | **3** | **2** | **6** | **16** | **46** | **10** | |
|  | **Types** | | | ***bla***  **EC** | ***bla*CTX-M-14** | | ***bla*CTX-M-55** | ***bla*CTX-M-64** | | ***bla*CTX-M-65** | ***bla*CTX-M-121** | ***bla*TEM-1** | ***bla*TEM-135** | ***bla*OXA-1** | ***bla*OXA-10** | ***bla*LAP-2** | ***bla*CMY-2** | | ***sul*1** | ***sul*2** | ***sul*3** | ***dfr*A1** | ***dfr*A5** | ***dfr*A8** | ***dfr*A12** | ***dfr*A14** | ***dfr*A17** |  |
| HeBei16 |  | | | * | |  | * |  |  | |  |  |  |  |  |  |  | |  | * |  |  |  |  |  |  |  |  |
| HeBei17 |  | | | * | |  | * |  |  | |  | * |  |  | * |  |  | |  | * |  |  |  |  |  | * |  |  |
| HeBei18 |  | | | * | |  | * |  |  | |  |  | * |  |  |  |  | |  | * |  |  |  |  |  | * |  |  |
| HeBei19 |  | | | * | |  | * |  |  | |  | * |  |  |  | * |  | |  |  | * |  | * |  | * | * |  |  |
| HeBei20 |  | | | * | |  | * |  |  | |  | * |  |  |  | * |  | |  | * | * |  |  | * |  | * |  |  |
| HeBei21 |  | | | * | |  |  |  | * | |  |  |  |  | * |  |  | |  |  | * |  |  |  |  |  |  |  |
| HeBei22 |  | | | * | |  |  |  | * | |  | * |  | * |  |  |  | | * | * |  |  |  |  | * |  | * |  |
| JiangSu1 | |  | | * | |  | * |  |  | |  | * |  |  |  | * |  | |  | * | * |  |  |  |  | * |  |  |
| JiangSu2 | |  | | * | |  | * |  |  | |  | * |  |  |  | * |  | |  | * | * |  |  |  |  | * |  |  |
| JiangSu3 | |  | | * | |  |  |  |  | |  | * |  |  |  |  |  | |  |  |  |  |  |  |  |  |  |  |
| JiangSu4 | |  | | * | |  |  |  |  | |  | * |  |  |  |  |  | |  |  | * |  |  | * |  |  |  |  |
| JiangSu5 | |  | | * | |  | * |  |  | |  | * |  |  | * | * |  | |  |  | * |  |  | * |  | * |  |  |
| JiangSu6 | |  | | * | |  | * |  |  | |  | * |  |  | * | * |  | |  |  | * |  |  | * |  | * |  |  |
| JiangSu7 | |  | | * | |  | * |  |  | |  | * |  |  |  | * |  | |  |  | * |  |  | * |  | * |  |  |
| JiangSu8 | |  | | * | |  | * |  |  | |  | * |  |  |  | * |  | |  | * | * |  |  | * |  | * |  |  |
| JiangSu9 | |  | | * | |  | * |  |  | |  | * |  |  |  | * |  | |  |  | * |  |  |  |  | * |  |  |
| JiangSu10 | |  | | * | |  | * |  |  | |  | * |  |  | * | * |  | |  |  | * |  |  |  |  | * |  |  |
| JiangSu11 | |  | | * | |  | * |  |  | |  | * |  |  |  |  |  | |  | * |  |  |  |  |  |  |  |  |
| JiangSu12 | |  | | * | |  | * |  |  | |  | * |  |  |  |  |  | |  | * |  |  |  |  |  |  |  |  |
| JiangSu13 | |  | | * | |  | * |  |  | |  | * |  |  |  |  |  | | * | * | * |  |  |  |  |  | * |  |
| JiangSu14 | |  | | * | |  | * |  |  | |  | * |  |  |  | * | * | |  | * | * |  |  |  |  | * |  |  |
| JiangSu15 | |  | | * | |  |  |  | * | |  | * |  |  | * |  |  | |  | * | * |  |  |  | * | * |  |  |
| JiangSu16 | |  | | * | |  | * |  |  | |  | * |  |  |  |  |  | |  | * |  |  |  |  |  |  |  |  |
| JiangSu17 | |  | | * | |  |  |  | * | |  | * |  |  | * |  | * | |  | * |  |  |  |  |  | * |  |  |
| JiangSu18 | |  | | * | |  | * |  |  | |  | * |  |  |  |  |  | |  | * |  |  |  |  |  | * |  |  |
| FuJian2 | |  | | * | |  |  |  |  | |  |  | * |  |  |  |  | |  | * |  |  |  |  |  |  |  |  |
| FuJian3 | |  | | * | |  |  |  | * | |  | * |  |  | * |  |  | |  | * | * |  | * |  | * |  |  |  |
| FuJian4 | |  | | * | |  |  |  |  | |  |  | * |  |  |  |  | |  | * |  |  |  |  |  |  |  |  |
| FuJian5 | |  | | * | |  | * |  |  | |  | * |  |  |  |  |  | |  | * |  |  |  |  |  |  |  |  |
| FuJian6 | |  | | * | |  | * |  |  | |  | * |  |  |  |  |  | |  | * |  |  |  |  |  |  |  |  |
| FuJian7 | |  | | * | |  |  |  | * | |  |  |  |  | * |  |  | |  |  | * |  |  |  |  | * |  |  |
| NingXia1 | |  | | * | |  | * |  |  | |  | * |  |  |  |  |  | | * | * |  |  |  |  | * | * |  |  |
| NingXia2 | |  | | * | |  | * |  |  | |  | * |  |  |  |  |  | | * | * |  |  |  |  | * |  |  |  |
| NingXia3 | |  | | * | |  | * |  |  | |  | * |  |  |  |  |  | | * | * |  |  |  |  | * | * |  |  |
| NingXia4 | |  | | * | |  | * |  |  | |  | * |  |  |  |  |  | | * | * |  |  |  |  | * | * |  |  |
| NingXia5 | |  | | * | |  | * |  |  | |  | * |  |  |  |  |  | |  | * |  |  |  |  |  | * |  |  |
| NingXia6 | |  | | * | |  | * |  |  | |  | * |  |  |  |  |  | |  | * |  |  |  |  |  | * |  |  |
| NingXia7 | |  | | * | |  | * |  |  | |  | * |  |  |  |  |  | |  | * |  |  |  |  |  | * |  |  |
| NingXia8 | |  | | * | | * | * |  |  | |  | * |  |  |  |  |  | | * | * | * |  |  |  |  |  |  |  |
| NingXia9 | |  | | * | |  | * |  |  | |  | * |  |  |  |  |  | |  | * |  |  |  |  |  | * |  |  |
| NingXia10 | |  | | * | |  | * |  |  | |  | * |  |  | * |  |  | |  | * |  |  |  |  |  | * |  |  |
| NingXia11 | |  | | * | |  | * |  |  | |  | * |  |  | * |  |  | |  | * |  |  |  |  |  | * |  |  |
| ShaanXi1 | |  | | * | |  | * |  |  | |  | * |  |  |  |  |  | |  |  | * |  |  |  | * |  |  |  |
| ShaanXi2 | |  | | * | | * |  |  |  | |  | * |  |  |  |  |  | |  |  | * |  |  |  |  | * |  |  |
| ShaanXi3 | |  | | * | |  | * |  |  | |  | * |  |  |  | * |  | |  | * | * |  |  |  |  | * |  |  |
| ShaanXi4 | |  | | * | |  | * |  |  | |  | * |  |  | * |  |  | |  | * |  |  |  |  |  | * |  |  |
| ShaanXi5 | |  | | * | |  | * |  |  | |  | * |  |  | * |  |  | |  | * |  |  |  |  |  | * |  |  |
| ShaanXi6 | |  | | * | |  | * |  |  | |  | * |  |  | * |  |  | |  | * |  |  |  |  |  | * |  |  |

| Strain | ARGs | | Aminoglycoside | | | | | | | | | | | | | | | | | | Colistin | |  | Tetracycline | | | | |
| --- | --- | --- | --- | --- | --- | --- | --- | --- | --- | --- | --- | --- | --- | --- | --- | --- | --- | --- | --- | --- | --- | --- | --- | --- | --- | --- | --- | --- |
|  | **Number** | | | **40** | **28** | **9** | **16** | **25** | | **19** | **44** | **1** | **4** | **41** | **55** | **64** | **28** | **25** | **1** | **19** | **3** | **4** | **1** | | **86** | **3** | **29** | |
|  | **Types** | | | ***aad***  **A1** | ***aad***  **A2** | ***aad***  **A5** | ***aad***  **A22** | ***aac(3)-IVa*** | | ***aac(3)-IId*** | ***aac(3)-IIe*** | ***aac(6’)-Ib3*** | ***aac(6’)-Ib-cr5*** | ***aph(3’)-Ia*** | ***aph(3”)- Ib*** | ***aph(6)-Id*** | ***aph(3’)- IIa*** | ***aph(4)-Ia*** | ***aph(6)-Ic*** | ***rmtB1*** | ***mcr-1*** | ***mcr-1.1*** | ***tet***  **(X4)** | | ***tet***  **(A)** | ***tet***  **(B)** | ***tet***  **(M)** |  |
| HuNan1 |  | | | * | * |  |  | | * |  |  |  |  | * |  |  |  | * |  |  | * |  |  | |  |  | * |  |
| HuNan2 |  | | | * | * |  |  | | * |  |  |  |  | * | * | * |  | * |  |  |  |  |  | | * |  |  |  |
| HuNan3 |  | | |  |  |  | * | | * |  |  |  |  | * | * | * |  | * |  |  |  |  |  | | * |  |  |  |
| HuNan4 |  | | |  |  |  |  | |  |  |  |  |  |  |  |  |  |  |  |  |  |  |  | |  |  |  |  |
| HuNan5 |  | | |  |  | * |  | |  |  |  |  |  |  | * | * |  |  |  |  |  |  |  | | * |  |  |  |
| HuNan6 |  | | |  |  | * |  | |  |  |  |  |  |  | * | * |  |  |  |  |  |  |  | | * |  |  |  |
| HuNan7 |  | | | * |  |  |  | |  |  |  |  |  |  |  |  |  |  |  |  |  |  |  | | * |  |  |  |
| HuNan8 |  | | |  |  | * |  | |  |  |  |  |  |  | * | * |  |  |  |  |  |  |  | | * |  |  |  |
| HuNan9 |  | | |  |  |  |  | | * |  |  |  |  | * | * | * |  | * |  |  |  |  |  | | * |  |  |  |
| HuNan10 | |  | | * |  |  |  | |  |  |  |  |  |  |  |  |  |  |  |  |  |  |  | | * | * |  |  |
| HuNan11 | |  | | * | * |  |  | | * |  |  |  |  | * |  |  |  | * |  |  | * |  |  | |  |  | * |  |
| HuNan12 | |  | | * | * |  |  | | * |  |  |  |  |  | * | * |  | * |  |  |  |  |  | | * |  |  |  |
| HuNan13 | |  | |  |  |  | * | | * |  |  |  |  | * | * | * |  | * |  |  |  |  |  | | * |  |  |  |
| HuNan14 | |  | |  |  |  |  | |  |  |  |  |  |  |  |  |  |  |  |  |  |  |  | |  | * |  |  |
| HuNan15 | |  | |  |  | * |  | |  |  |  |  |  |  | * | * |  |  |  |  |  |  |  | | * |  |  |  |
| HuNan16 | |  | |  |  |  |  | | * |  |  |  |  |  | * | * |  | * |  |  |  |  |  | | * |  |  |  |
| HuNan17 | |  | | * | * |  |  | | * |  |  |  |  | * |  |  |  | * |  |  | * |  |  | |  |  | * |  |
| HuNan18 | |  | | * |  |  |  | |  |  |  |  |  |  |  |  |  |  |  |  |  |  |  | | * | * |  |  |
| HuNan19 | |  | | * |  |  |  | |  |  |  |  |  |  |  |  |  |  |  |  |  |  |  | | * |  |  |  |
| HuNan20 | |  | |  |  |  |  | |  |  |  |  |  |  | * | * |  |  |  |  |  |  |  | | * |  |  |  |
| HuNan21 | |  | |  |  | * |  | | * |  |  |  |  |  |  |  |  | * |  |  |  |  |  | | * |  |  |  |
| HuNan22 | |  | |  |  |  |  | |  |  |  |  |  |  |  |  |  |  |  |  |  |  |  | |  |  |  |  |
| HeNan1 | |  | |  |  |  |  | |  |  |  |  |  |  |  |  |  |  |  |  |  | * |  | |  |  |  |  |
| HeNan2 | |  | |  |  |  |  | |  |  |  |  |  |  |  |  |  |  |  |  |  |  |  | | * |  |  |  |
| HeNan3 | |  | |  |  |  |  | |  |  |  |  |  |  |  |  | * |  |  |  |  | * |  | |  |  |  |  |
| HeNan4 | |  | |  |  |  |  | |  |  |  |  |  |  |  |  |  |  |  |  |  | * |  | |  |  |  |  |
| HeNan5 | |  | |  |  |  | * | |  |  |  |  |  |  | * | * | * |  |  |  |  | * | * | | * |  | * |  |
| HeNan6 | |  | |  |  |  |  | | * |  |  |  |  |  | * | * | * | * |  |  |  |  |  | | * |  | * |  |
| HeNan7 | |  | |  |  |  |  | |  |  |  |  |  |  |  |  | * |  |  |  |  |  |  | | * |  | * |  |
| HeNan8 | |  | |  |  |  |  | |  |  |  |  |  |  | * | * | * |  |  |  |  |  |  | | * |  | * |  |
| HeNan9 | |  | |  | * |  |  | |  |  |  |  |  |  |  |  |  |  |  |  |  |  |  | | * |  |  |  |
| HeNan10 | |  | |  |  |  |  | |  |  |  |  |  |  | * | * | * |  |  |  |  |  |  | | * |  | * |  |
| HeBei1 | |  | | * |  |  |  | |  |  | * |  |  |  |  |  |  |  |  |  |  |  |  | | * |  |  |  |
| HeBei2 | |  | | * |  |  |  | |  |  | * |  |  |  |  |  |  |  |  |  |  |  |  | | * |  |  |  |
| HeBei3 | |  | | * |  |  |  | |  |  | * |  |  |  |  |  |  |  |  |  |  |  |  | | * |  |  |  |
| HeBei4 | |  | | * |  |  |  | |  |  | * |  |  |  |  |  |  |  |  |  |  |  |  | | * |  |  |  |
| HeBei5 | |  | |  | * | * |  | | * |  |  |  |  |  |  |  |  | * |  |  |  |  |  | | * |  |  |  |
| HeBei6 | |  | | * |  |  |  | | * |  |  |  |  | * |  |  |  | * |  |  |  |  |  | | * |  |  |  |
| HeBei7 | |  | | * | * |  |  | | * |  |  |  |  | * |  |  |  | * |  |  |  |  |  | | * |  |  |  |
| HeBei8 | |  | |  |  | * |  | | * |  |  | * |  |  |  |  |  | * |  |  |  |  |  | | * |  |  |  |
| HeBei9 | |  | | * |  |  |  | |  |  |  |  |  |  |  |  |  |  |  |  |  |  |  | | * |  |  |  |
| HeBei10 | |  | | * | * |  |  | | * |  |  |  |  | * | * | * |  | * |  |  |  |  |  | | * |  |  |  |
| HeBei11 | |  | | * | * |  |  | | * | * |  |  | * | * | * | * | * | * |  |  |  |  |  | | * |  | * |  |
| HeBei12 | |  | |  | * |  |  | | * |  |  |  | * | * | * | * |  | * |  |  |  |  |  | | * |  | * |  |
| HeBei13 | |  | | * |  |  |  | |  |  |  |  |  | * |  |  |  |  |  |  |  |  |  | | * |  |  |  |
| HeBei14 | |  | | * | * |  |  | |  |  |  |  |  |  |  |  | * |  |  |  |  |  |  | | * |  |  |  |
| HeBei15 | |  | | * | * |  |  | |  |  |  |  |  |  | * | * | * |  |  |  |  |  |  | | * |  |  |  |

| Strain | ARGs | | | Aminoglycoside | | | | | | | | | | | | | | | | | | Colistin | |  | Tetracycline | | | | |
| --- | --- | --- | --- | --- | --- | --- | --- | --- | --- | --- | --- | --- | --- | --- | --- | --- | --- | --- | --- | --- | --- | --- | --- | --- | --- | --- | --- | --- | --- |
|  | **Number** | | | | **40** | **28** | **9** | **16** | **25** | | **19** | **44** | **1** | **4** | **41** | **55** | **64** | **28** | **25** | **1** | **19** | **3** | **4** | **1** | | **86** | **3** | **29** | |
|  | **Types** | | | | ***aad***  **A1** | ***aad***  **A2** | ***aad***  **A5** | ***aad***  **A22** | ***aac(3)-IVa*** | | ***aac(3)-IId*** | ***aac(3)-IIe*** | ***aac(6’)-Ib3*** | ***aac(6’)-Ib-cr5*** | ***aph(3’)-Ia*** | ***aph(3”)- Ib*** | ***aph(6)-Id*** | ***aph(3’)- IIa*** | ***aph(4)-Ia*** | ***aph(6)-Ic*** | ***rmtB1*** | ***mcr-1*** | ***mcr-1.1*** | ***tet***  **(X4)** | | ***tet***  **(A)** | ***tet***  **(B)** | ***tet***  **(M)** |  |
| HeBei16 | |  | | |  |  |  |  | |  |  |  |  |  |  | * | * |  |  |  |  |  |  |  | | * |  |  |  |
| HeBei17 | |  | | | * |  |  |  | |  |  |  |  |  |  | * | * | * |  |  |  |  |  |  | | * |  |  |  |
| HeBei18 | |  | | |  |  |  |  | |  |  |  |  |  | * | * | * | * |  |  |  |  |  |  | | * |  | * |  |
| HeBei19 | |  | | | * | * |  |  | |  | * |  |  |  | * |  | * |  |  |  | * |  |  |  | | * |  | * |  |
| HeBei20 | |  | | |  |  |  | * | |  | * |  |  |  |  |  | * |  |  |  | * |  |  |  | | * |  | * |  |
| HeBei21 | |  | | | * | * |  |  | | * |  |  |  |  | * | * | * |  | * |  |  |  |  |  | | * |  |  |  |
| HeBei22 | |  | | |  | * | * |  | | * |  |  |  | * |  |  |  |  | * |  |  |  |  |  | | * |  |  |  |
| JiangSu1 | | |  | |  |  |  | * | |  | * |  |  |  | * |  | * |  |  |  |  |  |  |  | | * |  |  |  |
| JiangSu2 | | |  | |  |  |  | * | |  | * |  |  |  | * |  | * |  |  |  |  |  |  |  | | * |  |  |  |
| JiangSu3 | | |  | |  | * |  |  | |  | * |  |  |  | * | * | * |  |  |  |  |  |  |  | | * |  | * |  |
| JiangSu4 | | |  | |  | * |  |  | |  | * |  |  |  | * | * | * |  |  |  |  |  |  |  | | * |  | * |  |
| JiangSu5 | | |  | | * |  |  |  | |  | * |  |  |  | * |  | * |  |  |  |  |  |  |  | | * |  | * |  |
| JiangSu6 | | |  | | * |  |  |  | |  | * |  |  |  | * |  | * |  |  |  |  |  |  |  | | * |  | * |  |
| JiangSu7 | | |  | |  |  |  | * | |  | * |  |  |  | * | * | * |  |  |  |  |  |  |  | | * |  | * |  |
| JiangSu8 | | |  | |  |  |  | * | |  | * |  |  |  | * |  | * |  |  |  |  |  |  |  | | * |  | * |  |
| JiangSu9 | | |  | |  |  |  |  | |  | * |  |  |  | * |  | * |  |  |  |  |  |  |  | | * |  |  |  |
| JiangSu10 | | |  | | * |  |  |  | |  | * |  |  |  | * |  | * |  |  |  |  |  |  |  | | * |  | * |  |
| JiangSu11 | | |  | |  |  |  | * | |  |  |  |  |  | * | * | * | * |  |  | * |  |  |  | | * |  |  |  |
| JiangSu12 | | |  | |  |  |  | * | |  |  |  |  |  | * | * | * | * |  |  | * |  |  |  | | * |  |  |  |
| JiangSu13 | | |  | | * |  | * |  | | * |  |  |  |  | * | * | * | * | * |  |  |  |  |  | | * |  |  |  |
| JiangSu14 | | |  | |  |  |  | * | |  | * |  |  |  | * | * | * |  |  |  |  |  |  |  | | * |  |  |  |
| JiangSu15 | | |  | | * | * |  |  | | * |  |  |  |  | * | * | * |  | * |  |  |  |  |  | | * |  |  |  |
| JiangSu16 | | |  | |  |  |  | * | |  |  |  |  |  | * | * | * | * |  |  | * |  |  |  | | * |  |  |  |
| JiangSu17 | | |  | | * |  |  |  | | * |  |  |  |  | * | * | * |  | * |  |  |  |  |  | | * |  |  |  |
| JiangSu18 | | |  | |  |  |  | * | |  |  |  |  |  | * | * | * | * |  |  | * |  |  |  | | * |  |  |  |
| FuJian2 | | |  | |  |  |  | * | |  |  |  |  |  |  | * | * |  |  |  |  |  |  |  | | * |  | * |  |
| FuJian3 | | |  | | * | * |  |  | |  |  |  |  |  |  | * | * |  |  |  |  |  |  |  | | * |  | * |  |
| FuJian4 | | |  | |  |  |  | * | |  |  |  |  |  |  | * | * |  |  |  |  |  |  |  | | * |  | * |  |
| FuJian5 | | |  | |  |  |  |  | |  |  |  |  |  |  | * | * | * |  |  |  |  |  |  | | * |  |  |  |
| FuJian6 | | |  | |  |  |  |  | |  |  |  |  |  |  | * | * | * |  |  |  |  |  |  | | * |  |  |  |
| FuJian7 | | |  | | * | * |  |  | | * |  |  |  |  | * | * | * |  | * |  |  |  |  |  | | * |  |  |  |
| NingXia1 | | |  | |  | * |  |  | |  | * |  |  |  | * | * | * |  |  |  | * |  |  |  | | * |  | * |  |
| NingXia2 | | |  | |  | * |  |  | |  | * |  |  |  | * | * | * |  |  |  | * |  |  |  | | * |  | * |  |
| NingXia3 | | |  | |  | * |  |  | |  | * |  |  |  | * | * | * |  |  |  | * |  |  |  | | * |  | * |  |
| NingXia4 | | |  | |  | * |  |  | |  | * |  |  |  | * | * | * |  |  |  | * |  |  |  | | * |  | * |  |
| NingXia5 | | |  | |  |  |  |  | |  |  |  |  |  |  | * | * | * |  |  | * |  |  |  | | * |  |  |  |
| NingXia6 | | |  | |  |  |  |  | |  |  |  |  |  |  | * | * | * |  |  | * |  |  |  | | * |  |  |  |
| NingXia7 | | |  | |  |  |  |  | |  |  |  |  |  |  | * | * | * |  |  | * |  |  |  | | * |  |  |  |
| NingXia8 | | |  | | * | * |  |  | | * |  |  |  | * |  | * | * | * | * | * | * |  |  |  | | * |  |  |  |
| NingXia9 | | |  | |  |  |  |  | |  |  |  |  |  |  | * | * | * |  |  | * |  |  |  | | * |  |  |  |
| NingXia10 | | |  | | * |  |  |  | |  |  |  |  |  |  | * | * | * |  |  | * |  |  |  | | * |  |  |  |
| NingXia11 | | |  | | * |  |  |  | |  |  |  |  |  |  | * | * | * |  |  | * |  |  |  | | * |  |  |  |
| ShaanXi1 | | |  | | * | * |  |  | |  |  |  |  |  | * |  |  |  |  |  |  |  |  |  | | * |  | * |  |
| ShaanXi2 | | |  | | * | * |  |  | |  |  |  |  |  |  |  |  |  |  |  |  |  |  |  | | * |  |  |  |
| ShaanXi3 | | |  | |  |  |  | * | |  | * |  |  |  | * | * | * |  |  |  |  |  |  |  | | * |  |  |  |
| ShaanXi4 | | |  | | * |  |  |  | |  |  |  |  |  |  | * | * | * |  |  |  |  |  |  | | * |  | * |  |
| ShaanXi5 | | |  | | * |  |  |  | |  |  |  |  |  |  | * | * | * |  |  | * |  |  |  | | * |  |  |  |
| ShaanXi6 | | |  | | * |  |  |  | |  |  |  |  |  |  | * | * | * |  |  | * |  |  |  | | * |  |  |  |

| Strain | ARGs | Quinolone | | | | | Rifampin | | | Fosfomycin | | | Chloramphenicol | | | | | | Lincosamide | | Oxyazolidinones | | Macrolide | |
| --- | --- | --- | --- | --- | --- | --- | --- | --- | --- | --- | --- | --- | --- | --- | --- | --- | --- | --- | --- | --- | --- | --- | --- | --- |
|  | **Number** | **7** | **7** | **55** | **2** | **1** | **29** | | **3** | | **21** | **2** | | **77** | **13** | **21** | **4** | **4** | **24** | **1** | | **1** | **4** | **32** |
|  | **Types** | ***oqxB*** | ***oqxA2*** | ***qnrS1*** | ***qnrS2*** | ***qnrS13*** | ***arr-2*** | | ***arr-3*** | | ***fosA3*** | ***fosA7.5*** | | ***floR*** | ***cmlA1*** | ***cmlA5*** | ***catA2*** | ***catB3*** | ***lnu(F)*** | ***lnu(G)*** | | ***poxtA*** | ***erm(B)*** | ***mph(A)*** |
| HuNan1 |  |  |  |  |  |  |  |  | * | |  | * | | * |  |  |  |  |  |  | |  |  |  |
| HuNan2 |  |  |  | * |  |  | * |  |  | |  | * | |  | * |  |  | * |  |  | |  |  |  |
| HuNan3 |  |  |  | * |  |  |  |  |  | |  | * | |  |  |  |  | * |  |  | |  |  |  |
| HuNan4 |  |  |  |  |  |  |  |  |  | |  |  | |  |  |  |  |  |  |  | |  |  |  |
| HuNan5 |  |  |  |  |  |  |  |  | * | |  |  | |  |  |  |  |  |  |  | |  | * |  |
| HuNan6 |  |  |  |  |  |  |  | |  | |  |  | |  |  |  |  |  |  |  | |  |  | * |
| HuNan7 |  |  |  |  |  |  |  | |  | |  |  | |  |  |  |  |  |  |  | |  |  |  |
| HuNan8 |  |  |  |  |  |  |  |  |  | |  |  | |  |  |  |  |  |  |  | |  | * |  |
| HuNan9 |  |  |  | * |  |  |  | |  | |  |  | | * |  |  |  |  |  |  | |  |  | * |
| HuNan10 |  |  |  | * |  |  | * |  |  | |  |  | |  | * |  |  |  |  |  | |  |  |  |
| HuNan11 |  |  |  |  |  |  |  |  | * | |  | * | | * |  |  |  |  |  |  | |  |  |  |
| HuNan12 |  |  |  |  |  |  |  |  |  | |  | * | | * |  |  |  |  |  |  | |  |  |  |
| HuNan13 |  |  |  | * |  |  |  |  |  | |  | * | |  |  |  |  | * |  |  | |  |  |  |
| HuNan14 |  |  |  |  |  |  |  |  |  | |  |  | |  |  |  |  |  |  |  | |  |  |  |
| HuNan15 |  |  |  |  |  |  |  | |  | | * |  | |  |  |  |  |  |  |  | |  |  | * |
| HuNan16 |  |  |  |  |  |  |  |  |  | |  | * | |  |  |  |  |  |  |  | |  |  |  |
| HuNan17 |  |  |  |  |  |  |  |  | * | |  | * | | * |  |  |  |  |  |  | |  |  |  |
| HuNan18 |  |  |  | * |  |  | * | |  | |  |  | |  |  | * |  |  |  |  | |  |  |  |
| HuNan19 |  |  |  | * |  |  | * | |  | |  |  | |  |  | * |  |  |  |  | |  |  |  |
| HuNan20 |  |  |  | * |  |  |  | |  | |  |  | | * |  |  |  |  |  |  | |  |  |  |
| HuNan21 |  |  |  |  |  |  |  | |  | |  |  | | * |  |  |  |  |  |  | |  |  | * |
| HuNan22 |  |  |  | * |  |  |  | |  | |  |  | |  |  |  |  |  |  |  | |  |  |  |
| HeNan1 |  |  |  |  |  |  |  |  |  | |  |  | |  |  |  |  |  |  |  | |  |  |  |
| HeNan2 |  |  |  | * |  |  |  |  |  | |  | * | |  |  |  |  |  |  |  | |  |  |  |
| HeNan3 |  |  |  |  |  |  |  | |  | | * |  | |  |  |  |  |  |  |  | |  |  |  |
| HeNan4 |  |  |  |  |  |  |  |  |  | |  |  | |  |  |  |  |  |  |  | |  |  |  |
| HeNan5 |  |  |  | * |  |  |  |  |  | |  | * | |  |  |  |  |  | * |  | |  |  |  |
| HeNan6 |  |  |  | * | * |  |  | |  | |  |  | | * |  |  |  |  |  |  | |  |  | * |
| HeNan7 |  |  |  | * |  |  |  |  |  | |  | * | |  |  |  |  |  |  |  | |  | * |  |
| HeNan8 |  |  |  | * |  |  |  |  |  | |  | * | |  |  |  |  |  |  |  | |  |  |  |
| HeNan9 |  |  |  | * |  |  | * | |  | |  |  | | * |  | * |  |  |  |  | |  |  |  |
| HeNan10 |  |  |  | * |  |  |  | |  | |  |  | | * |  |  |  |  |  |  | |  |  |  |
| HeBei1 |  |  |  |  |  |  |  |  |  | |  | * | |  |  |  |  |  |  |  | |  |  |  |
| HeBei2 |  |  |  |  |  |  |  |  |  | |  | * | |  |  |  |  |  |  |  | |  |  |  |
| HeBei3 |  |  |  |  |  |  |  |  |  | |  | * | |  |  |  |  |  |  |  | |  |  |  |
| HeBei4 |  |  |  |  |  |  |  |  |  | |  | * | |  |  |  |  |  |  |  | |  |  |  |
| HeBei5 |  |  |  | * |  |  |  |  | * | |  | * | |  |  |  |  |  |  |  | |  | * |  |
| HeBei6 |  |  |  |  |  |  |  |  | * | |  | * | |  |  |  |  |  |  |  | |  |  |  |
| HeBei7 |  |  |  |  |  |  |  |  | * | |  | * | | * |  |  |  |  |  |  | |  |  |  |
| HeBei8 |  |  |  | * |  |  |  |  | * | |  | * | |  |  |  |  |  |  |  | |  |  |  |
| HeBei9 |  |  |  | * |  |  | * |  |  | |  |  | |  | * |  |  |  |  |  | |  |  |  |
| HeBei10 |  |  |  | * |  |  |  |  |  | |  | * | | * |  |  |  |  |  |  | |  |  |  |
| HeBei11 |  |  |  | * |  |  | * |  |  | |  | * | |  | * |  | * |  |  |  | |  | * |  |
| HeBei12 |  |  |  | * |  |  |  | | * | |  |  | | * |  |  |  | * |  |  | |  |  |  |
| HeBei13 |  |  |  | * |  |  | * | |  | |  |  | | * |  | * |  |  |  |  | |  |  |  |
| HeBei14 |  |  |  | * |  |  |  | |  | |  |  | | * | * |  |  |  |  |  | |  |  |  |
| HeBei15 |  |  |  | * |  |  |  |  |  | |  | * | | * |  |  |  |  |  |  | |  | * |  |

| Strain | ARGs | | Quinolone | | | | | Rifampin | | | Fosfomycin | | | Chloramphenicol | | | | | | Lincosamide | | Oxyazolidinones | | Macrolide | |
| --- | --- | --- | --- | --- | --- | --- | --- | --- | --- | --- | --- | --- | --- | --- | --- | --- | --- | --- | --- | --- | --- | --- | --- | --- | --- |
|  | **Number** | | **7** | **7** | **55** | **2** | **1** | **29** | | **3** | | **21** | **2** | | **77** | **13** | **21** | **4** | **4** | **24** | **1** | | **1** | **4** | **32** |
|  | **Types** | | ***oqxB*** | ***oqxA2*** | ***qnrS1*** | ***qnrS2*** | ***qnrS13*** | ***arr-2*** | | ***arr-3*** | | ***fosA3*** | ***fosA7.5*** | | ***floR*** | ***cmlA1*** | ***cmlA5*** | ***catA2*** | ***catB3*** | ***lnu(F)*** | ***lnu(G)*** | | ***poxtA*** | ***erm(B)*** | ***mph(A)*** |
| HeBei16 | |  |  |  |  |  |  |  |  |  | |  | * | |  |  |  |  |  |  |  | |  |  |  |
| HeBei17 | |  |  |  |  |  |  | * |  |  | |  | * | |  | * |  |  |  |  |  | |  |  |  |
| HeBei18 | |  |  |  | * |  |  |  |  | * | |  | * | |  |  |  |  |  |  |  | |  |  |  |
| HeBei19 | |  |  |  | * |  |  | * |  | * | | * | * | | * |  |  |  | * |  |  | |  | * |  |
| HeBei20 | |  |  |  |  |  | * | * |  |  | |  | * | |  |  |  |  | * |  |  | |  | * |  |
| HeBei21 | |  |  |  | * |  |  |  |  |  | |  | * | |  | * |  |  | * |  |  | |  |  |  |
| HeBei22 | |  |  |  |  |  |  |  | | * | | * |  | | * |  |  |  | * |  |  | |  |  | * |
| JiangSu1 | |  |  |  | * |  |  | * | |  | |  |  | | * |  |  |  |  | * |  | |  |  |  |
| JiangSu2 | |  |  |  | * |  |  | * |  |  | |  | * | |  |  |  |  | * |  |  | |  |  |  |
| JiangSu3 | |  |  |  | * |  |  |  |  |  | |  | * | |  |  |  |  | * |  |  | |  |  |  |
| JiangSu4 | |  |  |  | * |  |  |  |  |  | |  | * | |  |  |  |  | * |  |  | |  |  |  |
| JiangSu5 | |  |  |  | * |  |  | * | |  | |  |  | | * |  | * |  |  | * |  | |  |  | * |
| JiangSu6 | |  |  |  | * |  |  | * | |  | |  |  | | * |  | * |  |  | * |  | |  |  | * |
| JiangSu7 | |  |  |  | * |  |  | * | |  | |  |  | |  |  |  |  |  | * |  | |  |  | * |
| JiangSu8 | |  |  |  | * |  |  | * | |  | |  |  | | * |  |  |  |  | * |  | |  |  |  |
| JiangSu9 | |  |  |  | * |  |  | * | |  | |  |  | | * |  |  |  |  |  |  | |  |  | * |
| JiangSu10 | |  |  |  | * |  |  | * |  |  | |  | * | |  | * |  |  | * |  |  | |  | * |  |
| JiangSu11 | |  |  |  |  |  |  |  | |  | | * |  | | * |  |  |  |  |  |  | |  | * |  |
| JiangSu12 | |  |  |  |  |  |  |  | |  | | * |  | | * |  |  |  |  |  |  | |  | * |  |
| JiangSu13 | |  |  |  |  |  |  |  | |  | | * |  | | * |  |  |  |  | * |  | |  |  | * |
| JiangSu14 | |  |  |  | * |  |  | * | |  | |  |  | |  |  |  |  |  | * |  | |  |  | * |
| JiangSu15 | |  |  |  | * |  |  | * |  |  | |  | * | |  | * |  |  | * |  |  | |  | * |  |
| JiangSu16 | |  |  |  |  |  |  |  |  | * | |  | * | |  |  |  |  |  |  |  | | * |  |  |
| JiangSu17 | |  |  |  | * |  |  | * |  |  | |  | * | |  | * |  |  | * |  |  | |  |  |  |
| JiangSu18 | |  |  |  |  |  |  |  |  | * | |  | * | |  |  |  |  |  |  |  | | * |  |  |
| FuJian2 | |  |  |  | * |  |  |  |  |  | |  | * | |  |  |  |  |  |  |  | |  | * |  |
| FuJian3 | |  |  |  | * |  |  |  |  |  | | * | * | | * |  |  |  |  |  |  | |  | * |  |
| FuJian4 | |  |  |  | * |  |  |  |  |  | |  | * | |  |  |  |  |  |  |  | |  | * |  |
| FuJian5 | |  | * | * |  |  |  |  |  |  | |  | * | |  |  |  |  |  |  |  | |  |  | * |
| FuJian6 | |  | * | * |  |  |  |  |  |  | |  | * | |  |  |  |  |  |  |  | |  |  | * |
| FuJian7 | |  |  |  | * |  |  | * | |  | |  |  | | * |  | * |  |  |  |  | | * |  |  |
| NingXia1 | |  |  |  | * |  |  |  |  |  | |  | * | |  |  | * |  | * |  |  | |  | * |  |
| NingXia2 | |  |  |  | * |  |  |  |  |  | |  | * | |  |  | * |  | * |  |  | |  | * |  |
| NingXia3 | |  |  |  | * |  |  |  |  |  | |  | * | |  |  | * |  | * |  |  | |  | * |  |
| NingXia4 | |  |  |  | * |  |  |  |  |  | |  | * | |  |  | * |  | * |  |  | |  | * |  |
| NingXia5 | |  | * | * |  |  |  |  |  |  | |  | * | |  |  |  |  |  |  |  | |  |  | * |
| NingXia6 | |  | * | * |  |  |  |  |  |  | |  | * | |  |  |  |  |  |  |  | |  |  | * |
| NingXia7 | |  | * | * |  |  |  |  |  |  | |  | * | |  |  |  |  |  |  |  | |  |  | * |
| NingXia8 | |  | * | * |  | * |  |  | * | * | |  | * | | * |  |  | * |  |  |  | |  | * | * |
| NingXia9 | |  | * | * |  |  |  |  |  |  | |  | * | |  |  |  |  |  |  |  | |  |  | * |
| NingXia10 | |  |  |  | * |  |  | * |  |  | |  | * | |  | * |  |  |  |  |  | |  |  |  |
| NingXia11 | |  |  |  | * |  |  | * |  |  | |  | * | |  | * |  |  |  |  |  | |  |  |  |
| ShaanXi1 | |  |  |  | * |  |  |  | |  | |  |  | | * | * |  |  |  |  |  | |  |  | * |
| ShaanXi2 | |  |  |  | * |  |  |  |  |  | |  | * | | * |  |  |  |  |  |  | |  |  |  |
| ShaanXi3 | |  |  |  | * |  |  | * |  |  | |  | * | |  |  |  |  | * |  |  | |  | * |  |
| ShaanXi4 | |  |  |  | * |  |  | * |  |  | |  | * | |  | * |  |  |  |  |  | |  |  |  |
| ShaanXi5 | |  |  |  |  |  |  | * |  | * | |  |  | |  | * |  |  |  |  |  | |  |  |  |
